# Edible Ultralong Organic Phosphorescent Maltodextrin with Different Dextrose Equivalents Values for Afterglow Visualizing the Quality of Tablets

**DOI:** 10.3390/ph19040565

**Published:** 2026-04-01

**Authors:** Zhijian Zhong, Haolong Xiong, Liangshan Ming, Yongmei Guan, Ailing Wen, Pengdi Cui, Caiyun Sun, Weifeng Zhu, Zhe Li

**Affiliations:** 1 Key Laboratory of Modern Preparation of Traditional Chinese Medicine, National Key Laboratory for the Modernization of Classical and Famous Prescriptions of Chinese Medicine, Institute for Advanced Study, Ministry of Education, Jiangxi University of Chinese Medicine, Nanchang 330004, China; zzj@crjz.com (Z.Z.); xionghaolong1@jxutcm.edu.cn (H.X.); jazmaster@163.com (L.M.); guanym2008@163.com (Y.G.); 18270907350@163.com (A.W.); 18648193560@163.com (C.S.); 2 China Resources Jiangzhong Pharmaceutical Group Co., Ltd., Nanchang 330096, China; cuipengdi@crjz.com; 3 Technology and Innovation Center of Jiangxi Traditional Chinese Medicine Manufacturing and Process Quality Control, Nanchang 330004, China

**Keywords:** maltodextrin, stimulus-responsive phosphorescent, room-temperature phosphorescent, tablet, non-destructive detection

## Abstract

**Background:** This study deeply explores the influence of different dextrose equivalents (DE) values on room-temperature phosphorescence (RTP) properties of maltodextrin (MD) and its luminescence mechanism. The potential applications of MD tablets in non-destructive detection for afterglow visualizing are also explored. **Methods**: MD tablets with different DE values were prepared to investigate their RTP properties and afterglow effects. MD tablets were validated for afterglow signals and phosphorescence lifetimes under varying environmental conditions. Additionally, the unique afterglow effect of MD was used to detect the uniformity of tablets. Theoretical calculations of MD monomers and dimers were performed using time-dependent density functional theory. **Results**: The results demonstrated that MD with different DE values exhibited RTP properties, with phosphorescence lifetimes from 186.91 to 618.85 ms. The afterglow signals and phosphorescence lifetimes of MD tablets were influenced by multiple environmental conditions, i.e., relative humidity, temperature, oxygen, ultraviolet light, etc. Based on the afterglow effect of the MD, it is possible to non-destructively detect the uniform tablet. MD is an RTP material regulated by its DE value. Its phosphorescence mechanism is governed by a clustering-triggered emission mechanism, which is dominated by the rich hydrogen bond network. The material’s stimuli-responsive properties and pronounced afterglow effect make it a potential application for non-destructive detection. **Conclusions**: This study not only investigates the stimulus-responsive behavior of MD but also discovers a common, safe, and edible stimulus-responsive RTP material. These findings provide a new method for non-destructive detection of drugs and reducing the potential pharmacological risks during production, storage, and transportation.

## 1. Introduction

Stimulus-responsive phosphorescent materials can adjust their luminescent properties in response to external stimuli, such as temperature, pressure, humidity, light, pH, molecular weight, etc., to control luminescent properties [1]. Owing to their long-lived excited states, large Stokes shifts, low cost, and ease of processing, room-temperature phosphorescent (RTP) materials have been widely applied in fields including anti-counterfeiting and information encryption, information storage, non-destructive testing, bioimaging, and sensing [2,3,4,5,6,7,8]. Compared with conventional fluorescent materials, ultralong RTP properties render the optical properties more readily detectable by the naked eye. Multiple signal channels enhance the complexity of information encryption and detection, providing richer information. Traditionally, these compounds usually require doping with aromatic groups, heavy atoms or heteroatoms to form highly conjugated structures that facilitate intersystem crossing (ISC). On the other hand, these RTP materials are currently predominantly aromatic compounds that suppress non-radiative transitions and stabilize excited triplet states by creating a rigid environment [9]. However, the application of these materials has been limited by complicated processing and purification requirements, high toxicity, and harsh synthesis conditions [10]. Therefore, there is an urgent need to explore RTP materials with low toxicity, biocompatibility and sustainability.

This non-conventional luminophore is characterized by extensive electron delocalization. Such unorthodox luminophores usually contain non-conjugated functional groups, such as ether (-O-), hydroxyl (-OH), halogen (-X), carbonyl (-C=O), carboxyl (-COOH), and related moieties [11]. Furthermore, the luminescence mechanism of these RTP materials is mainly rationalized by the clustering-triggered emission (CTE) mechanism. In the CTE mechanism, the clustering of non-conventional chromophores with lone pairs of *n* or *π* electrons facilitates electron communication. At the same time, this material generates extended electronic delocalization, which leads to enhanced conformational rigidity of the material [12]. As a result, such a clustering can readily form excited states. Fortunately, natural polysaccharides are rich in reactive groups such as hydroxyl, carboxyl, and amino groups. These groups promote a network of strong hydrogen and covalent bonds that provides a rigid molecular environment [13]. Therefore, polysaccharides are an important non-conventional luminescent material.

Maltodextrin (MD) is a polysaccharide-based carbohydrate powder derived from starch via acid or enzymatic hydrolysis. It primarily consists of glucose, maltose, maltotriose and other oligosaccharides [14]. These oligosaccharides are linked by α-1,4-glycosidic bonds, forming either linear or branched structures. MD products are classified based on their degree of hydrolysis indicated by the dextrose equivalents (DE) value, which directly determines the polymer chain length and the relative molecular weight [15]. The DE value ranges from 0 to 100. The higher the DE value, the greater the glucose content and the lower the average relative molecular weight. The DE value affects the hygroscopicity, viscosity, and glass transition temperature of MD [16,17]. Badin et al. [17] used MD with different DE values as a model matrix to investigate the relationship between techno-functional properties and particle surface properties. The results evidenced that the DE values were significantly correlated with the wetting and nanomechanical properties of MD, including glass transition, physicochemical properties and particle morphology. However, the effect of DE of MD on its RTP properties has not been investigated.

Herein, we report a series of edible excipients with non-conventional luminescence, i.e., MD with different DE values, which exhibit unique stimuli-responsive RTP properties. Our previous research has first proved that MD is a hardness- and humidity-responsive RTP material [18]. It has been successfully applied in the non-destructive detection of tablets. In this study, the responsive characteristics of MD were further deeply investigated. First, the DE value was identified as a key factor influencing the RTP properties of MD. Second, MD was prepared into tablets and subjected to various environmental conditions, including hardness, humidity, temperature, ambient air contact, vacuum-drying environment, and ultraviolet (UV) irradiation, to confirm its stimuli-responsive properties through the afterglow effect in combination with phosphorescence lifetime. In addition, an experiment was carried out to assess the uniformity of the tablets by afterglow signal analysis. Finally, the relationship between DE values and the RTP properties of MD was verified by simulations, including HOMO/LUMO and time-dependent density functional theory (TD-DFT) theoretical calculations. MD with different DE values as a stimulus-responsive RTP material deeply expands the applications of non-destructive detection.

## 2. Results and Discussion

### 2.1. Exploration of Phosphorescence Properties of MD

MD, as a proven polysaccharide-based RTP material, exhibits its RTP due to the abundant hydroxyl (-OH) and carbonyl (-C=O) functional groups within its molecular structure, which facilitate the formation of an extensive hydrogen bonding network [18]. Dai et al. [19] developed a chiral photonic film composed of cellulose nanocrystals and MD to address the inherent limitations of pristine CNC, such as brittleness, water sensitivity, and humidity-triggered reversibility. Owing to the MD structure, it carries out abundant hydrogen bonding interactions with CNC, which simultaneously enhance its mechanical properties, improve its humidity cycling resistance, and modulate its optical activity. These favorable properties render it a promising candidate for applications in the field of optical sensing. Moreover, MD groups have π-electrons and n-electrons inherent in the functional moieties, which facilitate spatial conjugation and extensive electron delocalization within the MD molecule. This active electronic delocalization could exhibit a quantifiable correlation MD with different DE values. Starch hydrolysis produces MD of varying molecular weights and low-molecular-weight polysaccharides, which is regulated by the DE value. Currently, there are many studies on RTP of polysaccharides. Yuan et al. systematically investigated the RTP properties of glycoconjugates, including d-+-glucose, d-+-xylose, d-+-fructose, and d-galactose [20,21]. These polysaccharide compounds exhibit similar structures to MD, establishing foundational work for exploring MD’s intrinsic phosphorescence mechanism [20]. In addition, the DE value affects various physical and chemical properties of MD, including molecular weight size, glass transition temperature, reconstruction coefficient, adhesion, etc. [17]. However, there is a lack of studies on the DE and RTP properties of MD. Therefore, the present experiment was conducted to investigate the phosphorescence properties of MD with different DE values.

**Figure 1:**
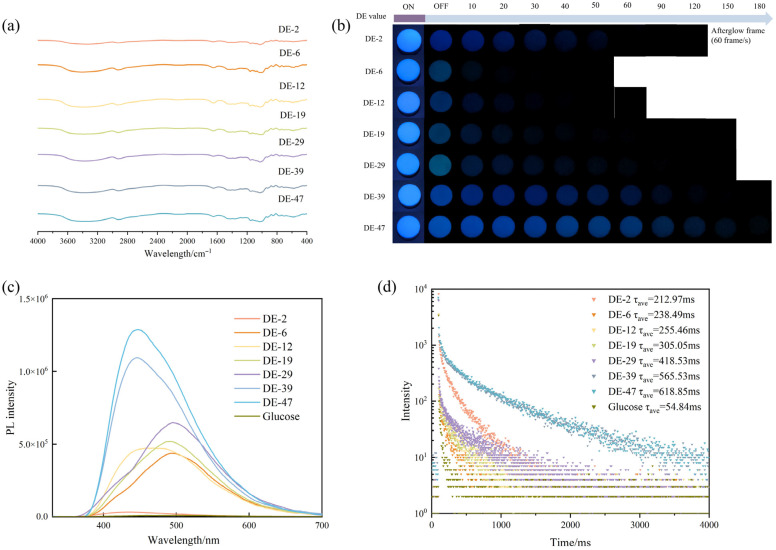
(**a**) Fourier transform infrared spectra of MD tablets with different DE values. (**b**) Afterglow photographs of MD tablets with different DE values. (**c**) Phosphorescence emission spectra of MD tablets with different DE values. (**d**) Phosphorescent lifetime decay of MD tablets with different DE values.

Firstly, the FTIR spectra of MD were explored to observe the functional groups of each sample (Figure 1a). All MD tablets had a characteristic strong O-H stretching vibration peak near 3317 cm^−1^, indicating that MD contained a large number of hydroxyl groups. Additionally, the C-H stretching vibration peak was observed at 2900 cm^−1^. MD showed a strong C=O stretching vibration peak at 1650 cm^−1^, which may undergo stretching vibrations and contribute to the formation of more stable triplet excited states. MD contains numerous hydroxyl and carbonyl groups, which form an extensive hydrogen bonding network and conjugated structure. These groups readily generate clustered trigger groups, promote spin–orbit coupling (SOC) and enhance rigid environments. In addition, MD originates from starch compounds, which are mainly composed of O and H atoms rich in lone electron pairs [22]. This composition indicates that the aggregation of O atoms and O-O contacts promotes electron overlap and spatial conjugation [12]. This type of structure of compounds lacks a significant conjugated luminescent unit, and they are therefore classified as exhibiting non-conventional luminescence, whose primary mechanism is CTE [13]. This RTP emission mechanism is similar for materials including nanocrystalline cellulose, bacterial cellulose, and sodium alginate, which are also rich in hydroxyl, carboxyl, or amino groups that can form an extensive network of hydrogen and covalent bonds, providing a rigid environment [23,24,25]. In the solid state, these materials tend to form dense cluster-triggered chromophores with stronger spatial interactions and more rigid bonding networks. Zhou et al. have widely reported that crystal powders can be compacted into tablets or films to enhance RTP properties [11,25]. Therefore, MD powders compacted into tablets represent an excellent strategy to enhance the RTP properties.

In order to better observe the afterglow effect and phosphorescence properties of MD, tablets with hardness of 100 N were prepared. Each tablet exhibited a significant phosphorescence emission effect, with a blue afterglow visible to the naked eye (Figure 1b). Besides phosphorescence lifetime, the gray value of afterglow imaging of tablets was also used to determine the afterglow intensity [18]. The afterglow imaging videos were imported into the video editing software ‘Cutcup’ for analysis. They were exported at 60 frames per second to ensure the standardization and comparability of afterglow photographs. This method was employed to compare the samples without an afterglow effect, which showed gray values of less than 1 × 10^−4^. Therefore, afterglow photographs with a gray value of less than 1 × 10^−4^ were regarded as without an afterglow effect. An afterglow photograph at this moment was defined as an afterglow frame by which the afterglow lifetimes can be determined. When the UV radiation was turned off, the fluorescence effect of the sample disappeared, exhibiting an afterglow that was generated by phosphorescence. Therefore, the first frame of the afterglow photograph after switching off the UV light was defined as the initial gray value, indicating the maximum afterglow intensity [18].

According to the DE values of MD (MD DE-2, MD DE-6, MD DE-12, MD DE-19, MD DE-29, MD DE-39, MD DE-47), the afterglow frames of MD tablets were 105.3, 28.6, 52, 80, 106, and 271.3, respectively (Table 1 and Figure 1b). The results showed that all the samples had a significant blue afterglow effect (Figure 1b). With the exception of MD DE-2, higher DE values corresponded to stronger afterglow effects. MD DE-6 exhibited the weakest afterglow effect, and MD DE-47 had the strongest afterglow effect with 3 s afterglow imaging visible to the naked eye. MD DE-2 displayed an afterglow effect between MD DE-19 and MD DE-29. Herein, we propose two hypotheses: (1) The phosphorescence emission behavior of MD and the afterglow effect might be directly correlated with glucose content. The higher the glucose content, the stronger the afterglow effect. (2) The phosphorescence emission of MD was dependent on its molecular weight. The higher the DE value of MD, the lower the average molecular weight of MD. The presence of glucose moieties within MD led to the exposure of additional hydroxyl groups, enabling hydrogen bond formation between MD and glucose. This interaction established a more extensive hydrogen bonding network, thereby enhancing the RTP effect. The enhanced RTP performance might be attributed to the influence of the flowability of amorphous maltooligosaccharides within the active pharmaceutical ingredient (API) matrix [26].

In order to deeply understand the photophysical properties of MD, we compared phosphorescence spectra of MD with different DE values and glucose. When excited by 254 nm UV light, the emission peak ranged from 435 nm to 495 nm [27] (Figure 1c). The phosphorescence spectra of MD tablets exhibited a red shift with the increase in DE value, and the maximum emission wavelengths were 435 nm, 490 nm, 488 nm, 482 nm, 495 nm, 445 nm, and 440 nm, respectively. In contrast, the peak maximum wavelength of glucose was 476 nm. Owing to the lowest percentage of glucose in MD DE-2, the phosphorescence spectrum of MD dominated the emission, while MD with different DE values showed a red shift by glucose. The high DE value of MD is molecular, and small molecular MD had higher matrix motility [28]. MD DE-2 had a maximum phosphorescence emission wavelength at 435 nm, while other hydrolysis products also influenced the emission wavelength of MD. However, MD DE-2 had the weaker luminescence intensity, which could be attributed to its long linear or branched structure with high molecular weight. Despite there being fewer molecular entities and emission centers in MD DE-2, it had highly twisted molecular structures and larger phosphorescence-emitting clusters, which inhibited radiative transitions and produced a more durable afterglow effect. Therefore, the maximum emission wavelengths of MD and glucose were determined to be 435 nm and 476 nm, respectively. To confirm the enhancement of the phosphorescence lifetime by MD tablets, phosphorescence properties of MD tablets with different DE values were investigated. With the exception of MD DE-2, both phosphorescence intensity and lifetimes increased gradually with increasing DE value (Table 1). The phosphorescence lifetimes of all MD tablets were 212.97 ms, 186.91 ms, 255.46 ms, 305.05 ms, 418.53 ms, 565.53 ms, 618.85 ms, respectively. In contrast, the phosphorescence lifetime of glucose was 52.84 ms (Figure 1d). The results of the afterglow effect of glucose demonstrated that the gray values of the afterglow photographs of glucose were all less than 1 × 10^−4^, indicating no afterglow that was visible to the naked eye. Therefore, the results suggested that glucose was not responsible for the MD afterglow, which was mainly attributed to MD itself. In this case, MD was hydrolyzed to form a more rigid environment owing to the large number of hydroxyls in its structure, creating a higher number of intermolecular hydrogen networks. On the other hand, the shorter contact distance between non-conventional luminescent materials was more favorable for the generation of phosphorescence [12,29]. Due to the intramolecular spatial blocking effect of MD, the molecular rotation was effectively suppressed, and the phosphorescence emission was stronger [30]. The phosphorescence lifetime of MD DE-2 was higher than that of DE-6. As the average molecular weight decreased, the phosphorescence lifetime of the MD with molecular rotationally suppressed emission decreased and transformed into phosphorescence emission dominated by the hydrogen network within MD. The RTP properties of MD provided rich specificity information for the stimuli-responsive behavior in non-destructive detection applications.

### 2.2. Pressure Response of MD

**Figure 2:**
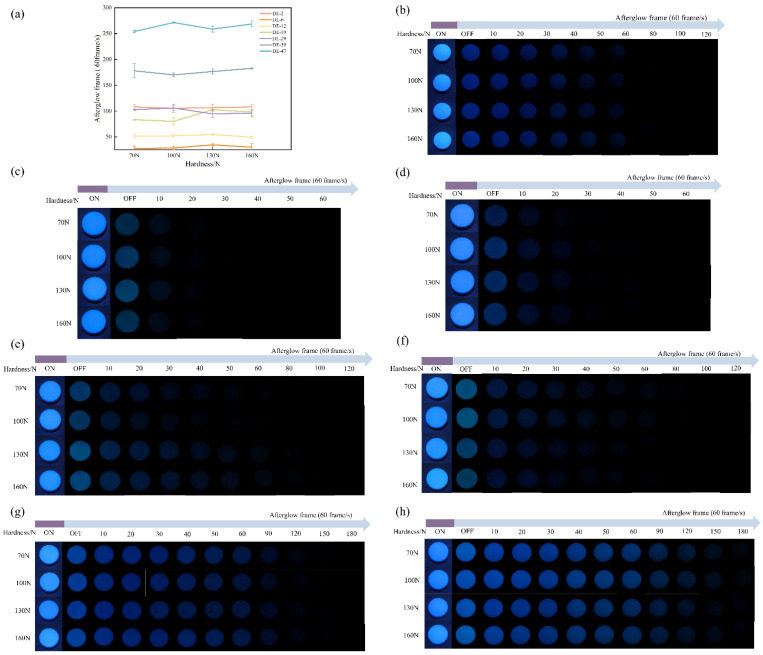
(**a**) Afterglow frames of MD tablets with different DE values under different hardnesses. (**b**–**f**) Afterglow photographs of MD tablets under different hardnesses; (**b**) DE-2, (**c**) DE-6, (**d**) DE-12, (**e**) DE-19, (**f**) DE-29, (**g**) DE-39, (**h**) DE-47.

The relationship between tablet pressure and the phosphorescence properties of MD was explored. Pressure and hardness showed a positive proportional correlation within a certain range. Consequently, this study employed hardness to represent pressure, thereby aligning more closely with practical applications. Tablets with hardness of 70, 100, 130 and 160 N were used for hardness response testing (Figure 2a). Subsequently, MD tablets were measured with afterglow photographs (Figure 2b–h). The results showed that the afterglow photographs of MD tablets with different hardnesses were indistinguishable to the naked eye. The afterglow frames for MD tablets with 70, 100, 130, and 160 N for DE-2 tablets were 108, 105.3, 106.6, and 108, respectively. Those for MD DE-6 tablets were 27.3, 28.6, 34.6, and 30.0, respectively. Those for MD DE-12 tablets were 51.3, 52.0, 54.6, and 49.3, respectively. Those for MD DE-19 tablets were 83.3, 80.0, 102.6, and 98.0, respectively. Those for MD DE-29 tablets were 102.6, 106.0, 94.6, and 96.0, respectively. Those for MD DE-39 tablets were 178.0, 170.0, 176.6, and 182.6, respectively. Those for MD DE-47 tablets were 254, 271.3, 258.6, and 268.6, respectively (Table 2). The afterglow frames of the MD DE-2 tablet were longer than those of MD DE-6, MD DE-12, MD DE-19 and MD DE-29, while shorter than those of MD DE-39 and MD DE-47. The afterglow frames increased with rising MD value for MD tablets except DE-2. However, it was difficult to distinguish the differences in afterglow photographs among MD tablets with different hardnesses. Owing to the CTE mechanism of MD, the increase in relative density of MD under pressure led to a reduction in average intermolecular spacing and enhanced the system’s rigid environment, thereby contributed to improved SOC [31]. On the other hand, the CTE mechanism of MD induces the molecular conformation to be rigid, reducing the energy dissipation of the non-radiative transitions. The compaction process improves the RTP ability of the samples by reducing the effect of triplet state quenchers and bringing molecules close together [25,32]. However, MD molecules in the amorphous state are disordered under external pressure, and the rigid environment was significantly weaker than that of crystalline compounds [33]. The phosphorescence emission of amorphous compounds is mainly due to vibrational stretching of the amorphous state, and it is less affected by external pressure compared with crystalline substances. Peng et al. [34] examined this phenomenon based on the properties of cellulose-based polymer CTE. It was found that a sodium carboxymethyl cellulose tablet enhanced through-space interaction and increased molecular packing density, which effectively suppressed non-radiative behavior. In the stress range of 132 to 461 MPa, the phosphorescence lifetime of the sodium carboxymethyl cellulose showed a significant pressure response.

### 2.3. Humidity Response of MD

**Figure 3:**
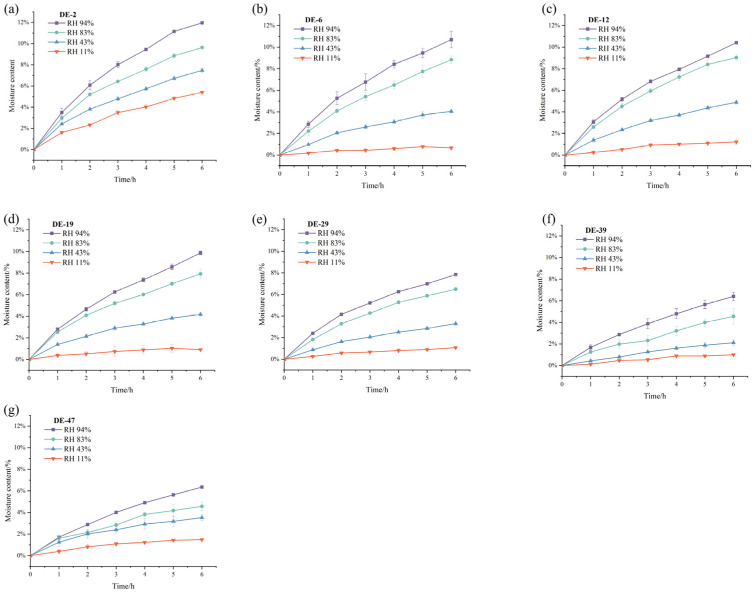
Hygroscopicity of MD tablets with different DE values stored in different relative humidity (RH) environments. (**a**) DE-2, (**b**) DE-6, (**c**) DE-12, (**d**) DE-19, (**e**) DE-29, (**f**) DE-39, (**g**) DE-47.

The MD tablets were stored in four different relative humidity (RH) environments of 94%, 83%, 43%, 11%, and the moisture contents of the tablets were weighed each hour (Figure 3a–g). After being stored in RH of 94%, 83%, 43%, and 11% for 6 h, MD DE-2 tablets gained moisture content by 11.39%, 9.41%, 6.84%, and 5.37%, respectively; MD DE-6 tablets gained moisture content by 10.98%, 9.40%, 4.78%, and 2.51%, respectively; MD DE-12 tablets gained moisture content by 10.69%, 9.03%, 5.77%, and 2.37%, respectively; MD DE-19 tablets gained moisture content by 9.84%, 7.11%, 4.22%, and 1.92%, respectively; MD DE-29 tablets gained moisture content by 7.84%, 6.49%, 3.29%, and 1.92%, respectively; MD DE-39 tablets gained moisture content by 6.40%, 4.54%, 2.11%, and 0.99%, respectively; MD DE-47 tablets gained moisture content by 6.35%, 4.56%, 3.52%, 1.48%, respectively (Table 3). It was found that the moisture content of the MD tablets increased gradually with rising environmental RH. At 94% RH, MD DE-2 tablets exhibited a maximum moisture content of 11.39%, while MD DE-47 tablets gained 6.35%. The maximum moisture content of the tablets decreased with increasing DE values. This could be attributed to the properties of MD, whereby the DE value influenced the hygroscopicity of MD tablets [15]. In addition, 94% RH environmental conditions were the main driving force for the glass transition of MD. The glass transition of MD DE-47 and MD DE-39 tablets stored in RH 94% and RH 83% environments is always accompanied by significant global surface smoothing. This surface smoothing phenomenon of MD DE-47 and MD DE-39 tablets was also accompanied by decrease in the tablet’s surface roughness. Previous studies indicated that, when the small molecule MD content of MD DE-39 and MD DE-47 increased, it was accompanied by higher hydroxyl group abundance and enhanced hydrogen bonding interactions. This promoted greater water contact, which was attributed to increased exposure of reducing ends. When a greater number of hydroxyls were exposed to the external surface of the molecule, it made MD tablets more easily absorb moisture and undergo glass transition [35]. However, Badin et al. [17] showed that the Guggenheim–Anderson–de Boer model fitted to the hygroscopic behavior of MD with high DE values stored in high-RH environments predicted a dramatic increase in water adsorption. A lower DE value was correlated with accelerated moisture absorption kinetics and enhanced hygroscopic capacity in molecular systems.

**Figure 4:**
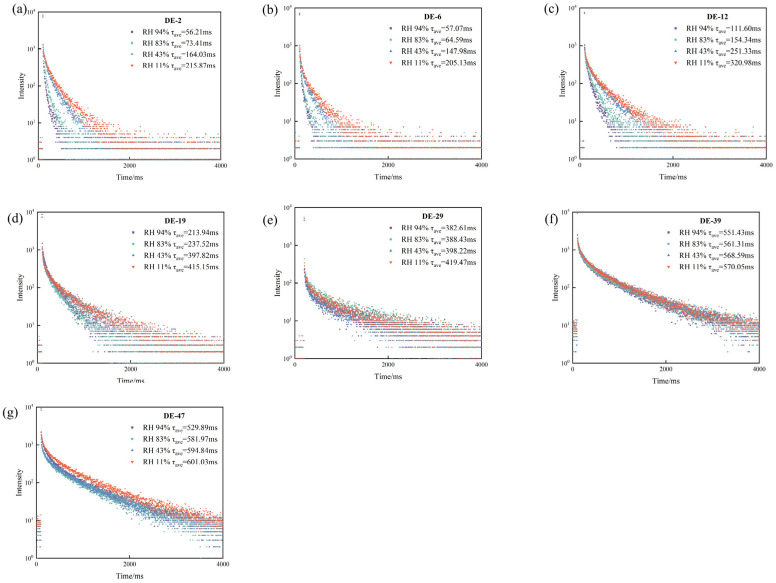
Phosphorescent lifetime decay of MD tablets with different DE values stored in different RH environments. (**a**) DE-2, (**b**) DE-6, (**c**) DE-12, (**d**) DE-19, (**e**) DE-29, (**f**) DE-39, (**g**) DE-47.

Moreover, we used a transient/steady state phosphorescence spectrophotometer to measure the phosphorescence lifetimes of the MD tablets under different moisture contents (Table 3). After 6 h of hygroscopicity testing at RH levels of 94%, 83%, 43%, and 11%, MD DE-2 tablets exhibited phosphorescence lifetimes of 56.21, 73.41, 164.03, and 215.87 ms, respectively; MD DE-6 tablets exhibited phosphorescence lifetimes of 57.07, 64.59, 147.98, and 205.13 ms, respectively; MD DE-12 tablets exhibited phosphorescence lifetimes of 110.60, 154.34, 251.33, and 320.98 ms, respectively; MD DE-19 tablets exhibited phosphorescence lifetimes of 213.94, 237.52, 397.82, and 415.15 ms, respectively; MD DE-29 tablets exhibited phosphorescence lifetimes of 382.61, 388.43, 398.22, and 419.47 ms, respectively; MD DE-39 tablets exhibited phosphorescence lifetimes of 551.43, 561.31, 568.59, and 570.05 ms, respectively; MD DE-47 tablets exhibited phosphorescence lifetimes of 529.89, 581.97, 594.84, and 601.03 ms, respectively (Figure 4a–g and Table 3). The results showed that there was a remarkable responsive relationship between the phosphorescence lifetime of MD tablets and moisture content. The average phosphorescence lifetime decreased as the moisture content of the tablets increased. The results indicate that water disrupts the hydrogen bond interactions between MD molecules. This leads to the depolymerization of the CTE groups, disrupting the rigid hydrogen bond network in the polysaccharide–polymer system. Meanwhile, water forms new hydrogen bonds with the MD molecules, resulting in the degrees of freedom of the molecules increasing [2]. The water quenches the triplet excitons, a process accelerating the decay of non-radiative transitions and decreasing the RTP properties of MD [36]. The afterglow signal and phosphorescence lifetime exhibited a consistent trend. Notably, MD DE-39 tablets and MD DE-47 tablets demonstrated ultralong phosphorescence lifetimes of 500–600 ms stored under different RH environments, accompanied by stronger resistance to moisture quenching. This capability of resisting moisture-induced phosphorescence quenching, coupled with persistent RTP properties, endowed the material with enhanced environmental responsiveness and provided richer information for non-destructive detection applications.

**Figure 5:**
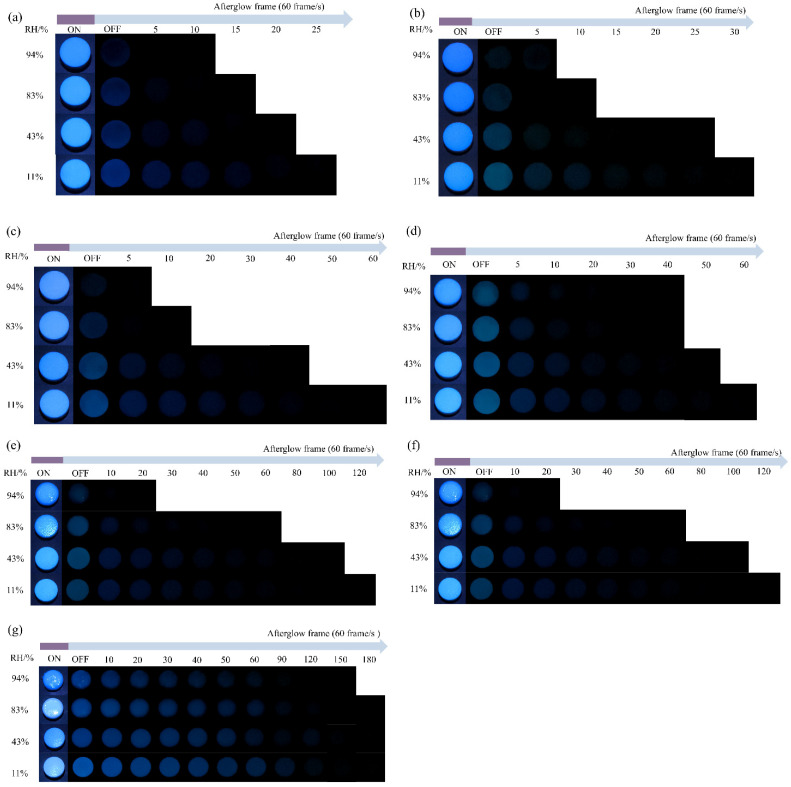
Afterglow photographs of MD tablets stored in different RH environments. (**a**) DE-2, (**b**) DE-6, (**c**) DE-12, (**d**) DE-19, (**e**) DE-29, (**f**) DE-39, (**g**) DE-47.

In addition, the relationship between the moisture content of tablets and the afterglow effect was also studied (Figure 5a–g). It was found that the afterglow signals of the tablets weakened with increasing moisture content. After being stored at RH levels of 94%, 83%, 43%, and 11% for 6 h, MD DE-2 tablets exhibited afterglow frames of 3.3, 11.3, 20.6, and 42.6, respectively; MD DE-6 tablets exhibited afterglow frames of 4.6, 7.3, 21.3, and 42.6, respectively; MD DE-12 tablets exhibited afterglow frames of 8.0, 9.3, 25.3, and 44.6, respectively; MD DE-19 tablets exhibited afterglow frames of 15.3, 19.3, 67.3, and 83.3, respectively; MD DE-29 tablets exhibited afterglow frames of 14.6, 54.0, 95.3, and 96.6, respectively; MD DE-39 tablets exhibited afterglow frames of 110.0, 170.0, 190.6, and 194.0, respectively; MD DE-47 tablets exhibited afterglow frames of 109.3, 124.0, 189.3, and 204.0, respectively (Table 4). These results revealed that the afterglow frames of tablets decreased with increasing moisture content. Notably, afterglow frames of MD DE-2, MD DE-6, and MD DE-12 exhibited minimal variations when stored in four RH conditions, suffering the most significant afterglow decay (Table 4). In contrast, MD DE-19 and MD DE-29 showed moderate moisture-induced quenching but still exhibited a visually observable afterglow. Remarkably, MD DE-39 and MD DE-47 demonstrated the weakest moisture susceptibility, maintaining prominent afterglow effects. It was related to the CTE mechanism of the DE tablet. Moisture content directly affects the intermolecular hydrogen bond network, causing the generation of new hydrogen bond networks and quenching the stability of the triplet excited state. MD tablets which were stored in 94% RH conditions absorbed a large amount of moisture, resulting in disruption of intermolecular electronic interactions and causing weakening of the ionic and hydrogen bonds, cluster disaggregation, and non-radiative transition enhancement. In addition, the afterglow frame of the MD DE-2 tablet was similar to that of the MD DE-29 tablet (Table 1). Owing to different DE values, the hygroscopic capacity of the MD DE-29 tablet was less than that of the MD DE-2 tablet, and the afterglow signal of the MD DE-29 tablet was better than that of the MD DE-2 tablet stored in four different RH environments. These findings indicated that MD DE-29 exhibited a higher resistance to moisture quenching when compared to DE-2. At RH 94% and 83%, DE-39 MD tablets and DE-47 MD tablets were susceptible to glass transition. However, this glass transition did not affect the CTE behavior of MD DE-39 tablets and MD DE-47 tablets. They exhibited an afterglow effect in four different RH environments, with an afterglow frame exceeding 100. This was significantly stronger than the afterglow effect of MD DE-2 tablets, MD DE-6 tablets, and MD DE-12 tablets after hygroscopicity testing (Table 4). Furthermore, the afterglow intensity of MD tablets was determined based on their initial gray values. Under RH 94%, 83%, 43%, and 11%, MD DE-2 tablets displayed initial gray values of 0.293, 0.845, 1.201, and 1.740, respectively; MD DE-6 tablets displayed initial gray values of 0.363, 0.514, 1.201, and 1.844, respectively; MD DE-12 tablets displayed initial gray values of 0.529, 0.650, 1.340, and 1.644, respectively; MD DE-19 tablets displayed initial gray values of 1.082, 1.853, 1.920, and 2.085, respectively; MD DE-29 tablets displayed initial gray values of 0.595, 1.172, 1.719, and 1.645, respectively; MD DE-39 tablets displayed initial gray values of 1.445, 2.081, 2.325, and 2.421, respectively; MD DE-47 tablets displayed initial gray values of 1.145, 1.574, 2.253, and 2.499, respectively (Table 4). Influenced by moisture, the afterglow signals of each MD tablet in different RH environments decreased significantly with increasing moisture content, which demonstrated the moisture-responsive RTP property of MD. In addition, the afterglow signals of the MD DE-47 tablet and MD DE-39 tablet were less affected by stronger resistance to moisture quenching. This capability of resisting moisture-induced phosphorescence quenching, coupled with persistent RTP properties, endowed the material with enhanced environmental responsiveness and provided more comprehensive information for non-destructive detection applications.

### 2.4. Temperature Response of MD

**Figure 6:**
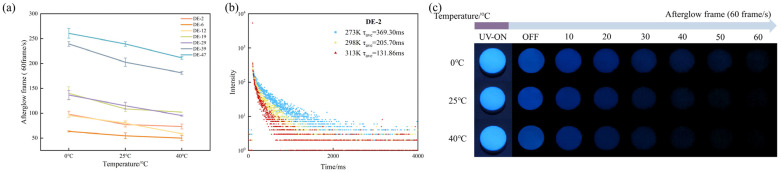
(**a**) Afterglow frames of MD tablets with different DE values stored at different temperatures. (**b**) Phosphorescent lifetime decay of DE-2 MD tablets stored at different temperatures. (**c**) Afterglow photographs of DE-2 MD tablets stored at different temperatures.

To systematically investigate the temperature dependence of RTP in MD tablets, we designed a thermal experiment to quantify the afterglow effect after storage in different thermal conditions. MD tablets were exposed to three different temperature conditions. At temperatures of 0 °C/273 K, 25 °C/293 K, and 40 °C/313 K, the afterglow frames for MD DE-2 tablets were 98.0, 77.3, and 73.3, respectively; those for MD DE-6 tablets were 63.3, 54.6, and 50, respectively; those for MD DE-12 tablets were 88.6, 80.0, and 58.6, respectively; those for MD DE-19 tablets were 136.6, 115, and 95.3, respectively; those for MD DE-29 tablets were 140.0, 108.6, and 102.0, respectively; those for MD DE-39 tablets were 239.3, 202.6, and 181.3, respectively; those for MD DE-47 tablets were 260.6, 239.3, and 211, respectively (Table 5 and Figure 6a). Moreover, the initial gray values for MD DE-2 tablets were 2.152, 1.763, and 1.949, respectively; those for MD DE-6 tablets were 1.556, 1.442, and 1.583, respectively; those for MD DE-12 tablets were 1.642, 1.643, and 1.533, respectively; those for MD DE-19 tablets were 1.797, 1.657, and 1.755, respectively; those for MD DE-29 tablets were 1.887, 1.7681, and 1.785, respectively; those for MD DE-39 tablets were 2.132, 2.280, and 2.197, respectively; those for MD DE-47 tablets were 2.208, 2.247, and 2.175, respectively (Table 5).

The afterglow frames significantly exhibited temperature dependence of the MD tablets in temperature testing. Compared to MD DE-2 tablets stored at 298 K and 313 K, the afterglow frames of MD DE-2 tablets observed at 273 K were enhanced by 10–20%, exhibiting observable differences in afterglow imaging. Specifically, lowering the temperature from 298 K to 273 K had an effect on afterglow frames, while the initial gray value of afterglow photographs remained stable. A decrease in temperature restricted molecular motion, including the suppressed vibration and rotation of the hydroxyl groups on the MD tablet. Furthermore, this facilitated efficient excimer emission by suppressing dissociation and non-radiative decay. The inhibition of depolymerization and non-radiative decay also contributed to enhanced RTP performance at low temperatures through efficient excitatory conjugate emission. As the temperature increased, the molecular motion of MD increased. In the non-radiative transition, the triplet excited states relaxed to the ground state, and the RTP emission and the afterglow lifetime decreased [29,37].

MD DE-2 tablets showed phosphorescence lifetimes of 369.30 ms at 273 K, 205.70 ms at 298 K, and 131.86 ms at 313 K, respectively (Figure 6b and Table 6). As typical properties of most RTP materials, the phosphorescence lifetime increased significantly with decreasing temperature. Previous studies indicated that a low temperature resulted in weaker thermal motion within molecules, lattice contraction, and enhanced intermolecular interactions. However, increasing the temperature could induce hydrogen bond dissociation and weaken rigidity within the polymer system, diminishing both the ability to isolate oxygen and vibrational relaxation suppression efficiency [38]. Furthermore, as MD is an amorphous material, it is impossible for it to undergo crystallization at low temperatures. Alibekov et al. [39] showed that MD does not have crystalline forms and remains amorphous after being freeze-dried. Therefore, the present results suggested that, when MD was subjected to low-temperature conditions, this mainly suppressed the non-radiative decay of the first excited triplet state and influenced RTP performance. The results implied that the triplet monomer dominated the RTP properties, while the triplet state remained unstable at room temperature. At low temperatures, the triplet states were stabilized due to restricted vibration and rotation of the molecules, enhancing RTP emission. Furthermore, higher temperatures lead to thermal activation effects, which promote increased molecular vibrations and rotations. This expands the pathways for energy dissipation in the material [40]. Temperature can also affect the energy gap of the material, modulating the ISC efficiency from the singlet to the triplet excited state and providing more triplet excitons for RTP emission [41]. Tablets with certain mechanical strength also exhibited significant afterglow effects and phosphorescence properties (Figure 6c), offering potential applications for non-destructive detection of tablets [41].

### 2.5. Photostability of MD

**Figure 7:**
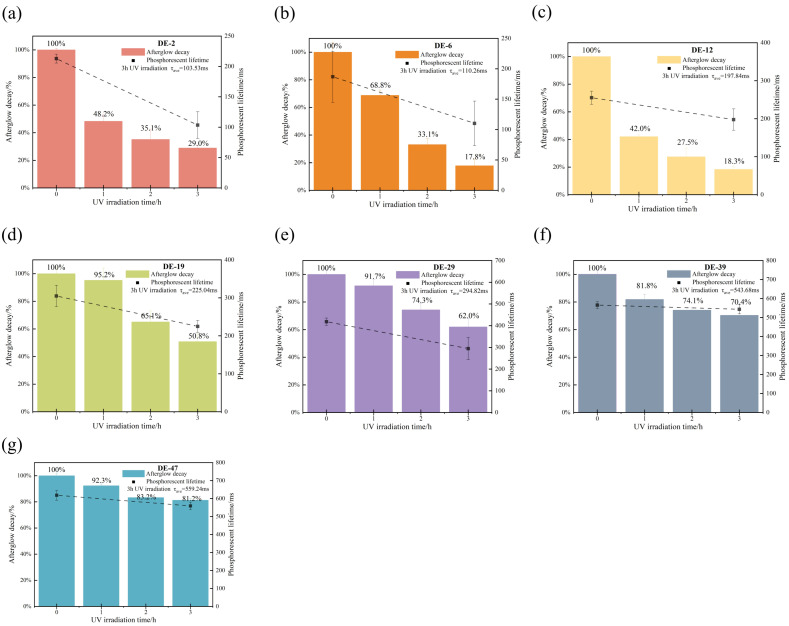
Afterglow decay of MD tablets with different DE values every hour and phosphorescent lifetime of MD tablets after UV irradiation of 3 h. (**a**) DE-2, (**b**) DE-6, (**c**) DE-12, (**d**) DE-19, (**e**) DE-29, (**f**) DE-39, (**g**) DE-47.

To assess the photostability of MD tablets with different DE values, we examined the effects of prolonged UV light irradiation on their afterglow and phosphorescence lifetimes. Both afterglow and phosphorescence lifetimes changed with extended UV light irradiation time [42]. After being subjected to UV irradiation for 3 h, MD DE-2 tablets exhibited an afterglow frame of 28.0, MD DE-6 tablets exhibited an afterglow frame of 9.3, MD DE-12 tablets exhibited an afterglow frame of 16.0, MD DE-19 tablets exhibited an afterglow frame of 64.0, MD DE-29 tablets exhibited an afterglow frame of 74.0, MD DE-39 tablets exhibited an afterglow frame of 163.3, and MD DE-47 tablets exhibited an afterglow frame of 220 (Table 7). The results demonstrated that the phosphorescence lifetimes of all MD tablets with different DE values decreased progressively with prolonged UV light irradiation [43]. Notably, MD DE-2 tablets, MD DE-6 tablets and MD DE-12 tablets demonstrated weak photostability after 3 h of UV light irradiation. The afterglow decay of MD DE-2 tablets, MD DE-6 tablets and MD DE-12 tablets retained 29.0%, 17.8%, and 18.3%, respectively (Figure 7a–c). MD DE-19 tablets and MD DE-29 tablets showed moderate photostability. The afterglow decay of MD DE-19 tablets and MD DE-29 tablets retained 50.8% and 62.0%, respectively (Figure 7d,e). Remarkably, MD DE-39 tablets and MD DE-47 tablets achieved optimal photostability and retained afterglow decay of 70.4% and 81.2%, respectively (Figure 7f,g). After 3 h of UV exposure, all tablets with different DE values maintained a visible afterglow effect over time (Figures S1–S3). With increasing DE value of MD tablets, the photostability gradually increased. Specifically, MD DE-39 tablets and MD DE-47 tablets exhibited superior photostability and UV resistance, as evidenced by afterglow decay of MD tablets under UV irradiation. Moreover, we particularly focused on the phosphorescence lifetime of MD tablets after prolonged UV light irradiation. It was clearly revealed that the phosphorescence lifetimes were reduced compared with the normal tablets. The phosphorescence lifetimes of MD tablets with increasing DE value after 3 h of prolonged UV light irradiation were 103.53 ms, 110.26 ms, 197.84 ms, 394.93 ms, 294.82 ms, 543.68 ms, and 559.24 ms, respectively (Figure 7 and Table 7). Compared to the standard MD tablets, the MD DE-2 tablets showed the most significant phosphorescence lifetime decay when exposed to prolonged UV light irradiation, while MD DE-47 tablets showed the least amount of phosphorescence lifetime decay. Prolonged UV light irradiation causes degradation of the hydrogen bond network and non-canonical conjugated structures in covalently crosslinked MD materials, resulting in decreased environmental rigidity and accelerated radiative transition [42]. In addition, prolonged exposure to UV light intensifies the molecular motion of MD, enhancing the non-radiative transitions of triplet excitons [19]. When subjected to short-term UV light, molecular oxygen in the mechanism quenches the triplet exciton, converting it into singlet oxygen. As the oxygen concentration decreases, triplet exciton quenching is suppressed, and the RTP emission is activated. Continuous accumulation of singlet oxygen may induce the oxidative degradation of the phosphorescence emission group. A shortened phosphorescence lifetime and a quenched afterglow effect are possible [44]. Both the phosphorescence lifetime and afterglow signal results showed that the phosphorescence emission of tablets was reduced to different degrees after prolonged UV light irradiation. This demonstrated that MD exhibited durable photostability. Compared to prolonged 3 h UV irradiation, the 10 s UV exposure protocol has a minimal effect on the afterglow, demonstrating its significant potential for practical applications.

### 2.6. Oxygen Response of MD

**Figure 8:**
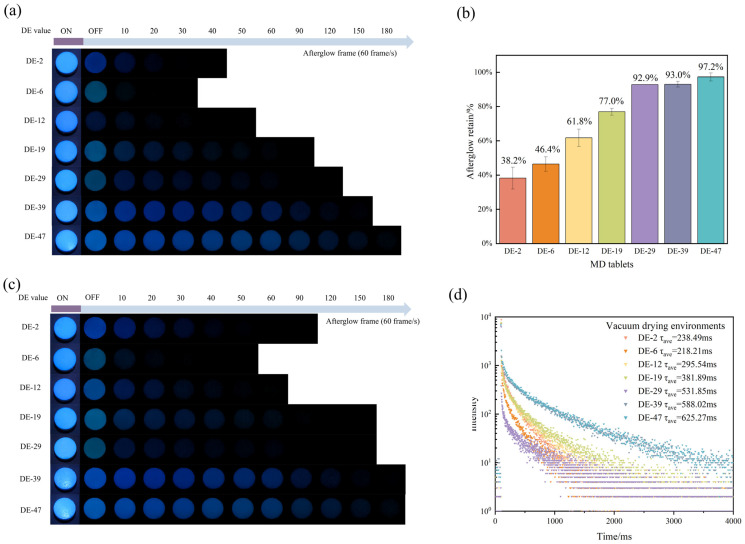
(**a**) Afterglow photographs of MD tablets with different DE values stored in ambient air contact environment. (**b**) Afterglow retention of MD tablets with different DE values stored in ambient air contact environment. (**c**) Afterglow photographs of MD tablets with different DE values stored in vacuum-drying environment. (**d**) Phosphorescent lifetime decay of MD tablets with different DE values stored in vacuum-drying environment.

The impact of ambient air on the phosphorescence lifetimes of the MD tablets with different DE values was evaluated. Additionally, comparative experiments were conducted via vacuum drying to eliminate air effects on the MD tablet. When subjected to vacuum drying and ambient air at 25 °C, MD DE-2 tablets exhibited afterglow frames of 65.3 and 34, respectively; MD DE-6 tablets exhibited afterglow frames of 40.0 and 22.0, respectively; MD DE-12 tablets exhibited afterglow frames of 82.0 and 50.6, respectively; MD DE-19 tablets exhibited afterglow frames of 103.3 and 96, respectively; MD DE-29 tablets exhibited afterglow frames of 116 and 89.3, respectively; MD DE-39 tablets exhibited afterglow frames of 202 and 188, respectively; MD DE-47 tablets exhibited afterglow frames of 244 and 238.6, respectively (Table 8). After being exposed to air for 3 h, the afterglow imaging of MD tablets with different DE values was retained (Figure 8a,c). The afterglow retentions of MD DE-2, DE-6, DE-12, DE-19, DE-29, DE-39, DE-47 tablets were 38.2%, 46.4%, 61.8%, 77.0%, 92.9%, 93.0%, and 97.2%, respectively (Figure 8b), exhibiting an upward trend with increasing DE values. The observed afterglow frames’ quenching under ambient air contact conditions was likely attributable to oxygen. Oxygen, a prevalent triplet state phosphorescence quencher, has two unpaired electrons in its ground state. This unique electronic structure enables efficient triplet energy transfer between oxygen and the triplet excited states of RTP materials, promoting non-radiative decay through spin-allowed electron exchange mechanisms [45]. This energy exchange mechanism allows oxygen to quench triplet excitons by absorbing their excitation energy, suppressing radiative phosphorescence emission. This result highlighted the remarkable stability of MD DE-29 tablets, MD DE-39 tablets, and MD DE-47 tablets in ambient air, with an afterglow retention rate above 90%. This phenomenon might be linked to the hydrolysis degree of MD. Hydrolysis levels of MD during industrial processing led to increased yields of low-molecular-weight oligosaccharides and glucose [28]. These smaller oligosaccharides and glucose also had RTP properties and were more resistant when subjected to oxygen quenching. Zang et al. [46] showed that materials with high sensitivity to oxygen exhibited quenching of phosphorescence under an oxygen environment. Glucose was an oxygen quencher in the high DE value system and facilitated efficient depletion of oxygen within the matrix. Moreover, this outstanding stability could also be attributed to the hydrogen bond network formed between the MD and the surrounding matrix. This framework created a protective environment that enhanced resistance to oxygen and moisture, factors that contribute to the degradation of phosphorescent materials [42].

The results showed that the phosphorescence lifetime was enhanced after storage under vacuum-drying conditions. The phosphorescence lifetimes of MD DE-2, DE-6, DE-12, DE-19, DE-29, DE-39, and DE-47 tablets were 238.49 ms, 218.21 ms, 295.54 ms, 381.89 ms, 531.85 ms, 588.02 ms, and 625.27 ms, respectively (Figure 8d and Table 9). All of them were longer than those of the tablets under ambient conditions. In a vacuum environment, the moisture removal mechanism relied on rapid evaporation from the tablet surface. However, this process created a hardened superficial layer that restricted internal moisture diffusion, ultimately resulting in residual moisture content within the tablet. The vacuum-drying environment exhibits enhanced drying efficiency, which allows for rapid sublimation or evaporation of moisture within the tablet. Its environment not only removed free water but also removed bound water from the material by low-temperature sublimation. Therefore, moisture within the tablets could be further reduced under vacuum to enhance the phosphorescence lifetime. The hydrogen bond networks within the MD, the oxygen quencher, smaller oligosaccharides, and glucose are essential for preventing the loss of phosphorescence. It thus ensured the photostability and suitability of these materials as phosphorescence-based environmental probes.

### 2.7. Application

Inspired by stimulus-responsive RTP materials, MD functions, as a phosphorescent probe, enabled non-destructive detection of tablets via afterglow imaging. Therefore, MD-based responsive probes could be engineered for quality monitoring and effectively reduce the risks from hygroscopic deterioration, oxidative degradation, and potential medicine risks.

**Figure 9:**
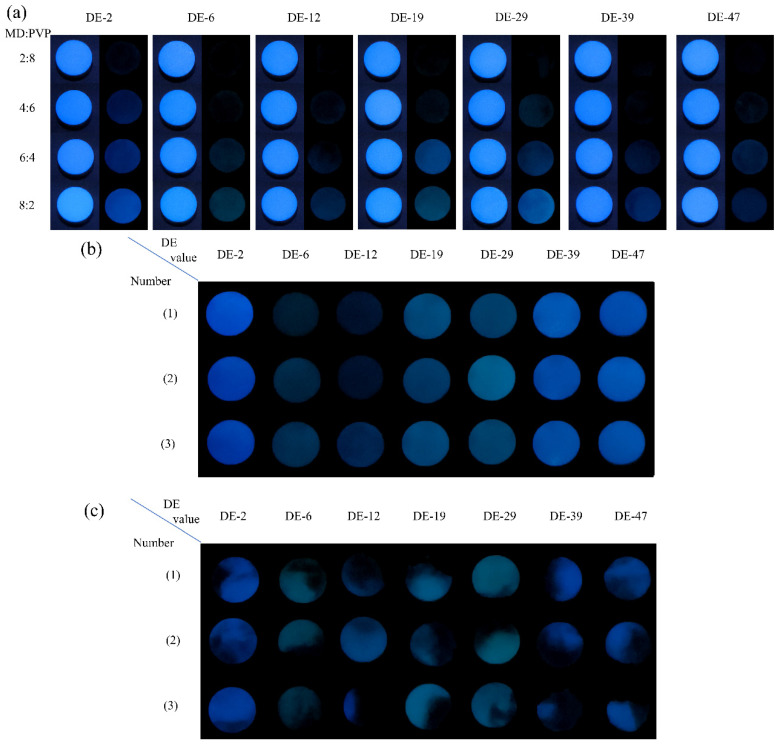
(**a**) Afterglow photographs of MD tablets with different mixing ratios of PVP and MD with different DE values. (**b**) Afterglow photographs of uniform tablets with different DE values. (**c**) Afterglow photographs of non-uniform tablets with different DE values.

The durations of the afterglow signal of tablets containing MD and PVP K30 (2:8, 4:6, 6:4, 8:2, *w*/*w*) with 100 N were determined under ambient environment conditions (Figure 9a). The afterglow effect of tablets with normal samples and different DE values should be established for a specified afterglow frame, i.e., the afterglow frame for MD DE-2 tablets is approximately 105.3. During the detection of afterglow photographs, the signal-to-background ratio in afterglow imaging was 50 times higher than that of autofluorescence interference owing to the elimination of the influence of the background signal [34]. This method greatly enhanced the sensitivity of tablet detection. In addition, the stimuli-responsive RTP properties of MD enable differentiation of tablets exposed to environmental conditions from those affected by production, packaging and transportation.

Furthermore, the spatial distribution characteristics of the afterglow effect on tablet surfaces could be utilized for analysis tablet information. Recent advances in UV irradiation coupled with machine learning algorithms have enabled direct quantification of API content and particle size distribution through afterglow imaging, which has garnered significant attention in real-time tablet quality monitoring [47]. Under UV irradiation, the optical characteristics of API particles were determined by surface smoothness and light reflectance. However, this direct UV detection method was susceptible to ambient light interference, affecting the signal-to-background ratio [34]. On the contrary, afterglow imaging exhibited significantly suppressed background signals compared to fluorescence modalities, resulting in much higher signal-to-background ratio. The results demonstrated that all MD samples formulated with PVP K30 at a mixing ratio of 2:8 exhibited no observable persistent afterglow, indicating complete suppression of the afterglow effect in this optical ratio (Figure 9a). It was demonstrated that the incorporation of a high percentage of PVP K30 into the MD facilitated a coating effect on the cluster emission groups. The coating effect masked the intrinsic afterglow emission properties of MD. Furthermore, the white PVP K30 layer could scatter and absorb light during transmission, leading to afterglow signals that complicated the observation of afterglow photographs. At a mixing ratio of 4:6 between MD and PVP K30, only the MD DE-2 tablets exhibited a distinct tablet outline in afterglow imaging, whereas MD with other DE values displayed a blurred outline under UV light excitation conditions. When the mixing ratio of MD to PVP K30 reaches 6:4, the tablet outline is clearly observed. This phenomenon should be attributed to the MD DE-2 phosphorescence exhibiting the shortest maximum emission wavelength of 435 nm. A shorter wavelength emission demonstrated superior transmittance through the tablet, facilitating phosphorescent emission to the tablet surface. These results indicate that the MD had a significant influencing effect on the RTP properties of the materials. MD DE-2 required 40% of MD excipients to exhibit a visible afterglow effect, while other samples depend on an optimal mixing ratio for PVP K30. Therefore, the uniformity of the tablets with the optimal ratio was assessed by analyzing the afterglow information on the surface of the tablets and comparing it with that of the non-uniform tablets.

The complete tablet outline was captured during the afterglow photograph analysis of uniformity of tablets (Figure 9b). To ensure the accuracy of a tablet’s uniformity analysis, it was performed on the outline of the tablet rather than the entire afterglow photograph. Therefore, this experiment compares the RSD values of uniform tablets to those of non-uniform tablets in order to clearly distinguish the uniformity of the tablets. The RSDs of the afterglow photographs of uniform MD DE-2 tablets were 7.21%, 6.37%, 7.19%, respectively; those of uniform MD DE-6 tablets were 20.64%, 10.80%, 8.27%, respectively; those of uniform MD DE-12 tablets were 13.09%, 15.00%, 8.37%, respectively; those of uniform MD DE-19 tablets were 12.76%, 14.38%, 5.52%, respectively; those of uniform MD DE-29 tablets were 7.08%, 6.60%, 8.43%, respectively; those of uniform MD DE-39 tablets were 4.97%, 3.91%, 5.38%, respectively; those of uniform MD DE-47 tablets were 8.34%, 6.13%, 6.39%, respectively (Table 10). However, through evaluating the RSD of non-uniform tablets, RSD values of the afterglow photographs of non-uniform MD DE-2 tablets were 42.00%, 35.79%, and 22.87%, respectively; those of non-uniform MD DE-6 tablets were 45.47%, 64.77%, and 70.05%, respectively; those of non-uniform MD DE-12 tablets were 72.40%, 21.16%, and 131.03%, respectively; those of non-uniform MD DE-19 tablets were 68.44%, 68.37%, and 69.95%, respectively; those of non-uniform MD DE-29 tablets were 35.52%, 63.72%, and 31.35%, respectively; those of non-uniform MD DE-39 tablets were 75.36%, 82.10%, and 92.95%, respectively; those of non-uniform MD DE-47 tablets 29.48%, 55.39%, and 90.53%, respectively (Table 11). Comparing to Figure 9b, Figure 9c, it shows that tablet uniformity is closely correlated with the relative standard deviation (RSD) of the afterglow photographs. The RSD values of afterglow photographs of the non-uniform tablets were more than 20% (Table 10). All afterglow photographs of uniform tablets exhibited a uniform afterglow distribution, and the RSD values of the afterglow photographs of the tablets were all less than 20%. Notably, the RSD values of afterglow photographs of MD DE-2 (3) and MD DE-12 (2) tablets were 22.87% and 21.16%, respectively. The afterglow photographs of these two tablets showed a distinct tablet afterglow outline, and the afterglow photographs exhibited partial non-uniformity. Meanwhile, the afterglow photographs of the uniform tablets exhibited a bright blue afterglow, and the RSD values of the afterglow photographs of these tablets were less than 20%. Therefore, the uniform tablets can be judged by the RSD of the afterglow photographs. This method for detecting uniform tablets was convenient and provided a new idea for non-destructive detection.

### 2.8. Theoretical Calculation

**Figure 10:**
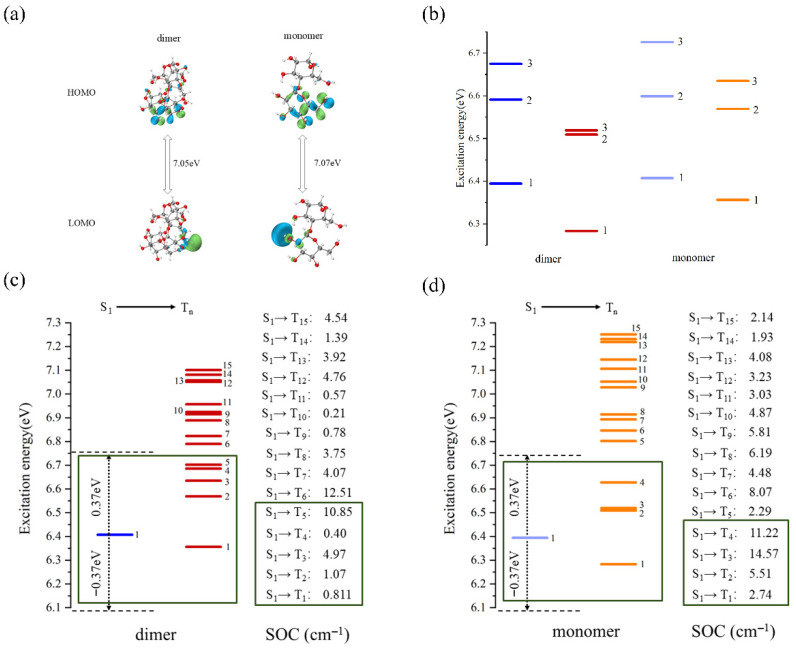
(**a**) HOMO and LUMO molecular orbitals of dimer and monomer of MD. (**b**) Energy levels of dimer and monomer of MD. (**c**) TD-DFT-calculated energy level diagram and the corresponding SOC constants of dimer. (**d**) TD-DFT-calculated energy level diagram and the corresponding SOC constants of monomer.

The phosphorescence mechanism of MD was further examined by theoretical simulation and data analysis. Experimentally, MD with varying degrees of hydrolysis was categorized into MD monomers and MD dimers. Based on the relationship between DE values and average molecular weight [35], MD monomers and dimers were regarded as MD DE-47 and MD DE-29, respectively. First, density functional theory (DFT) simulations were conducted to confirm the luminescence mechanism. Analysis of the HOMO/LUMO energy levels revealed that the dimer exhibits a more compact electron density distribution than the monomer, along with enhanced electrostatic attraction interactions resulting from orbital symmetry matching and charge redistribution. Twisted molecular configurations cause the HOMO to be primarily distributed along the main chain, while the LUMO is mainly localized on the branch chain [48]. Therefore, small energy gaps of the HOMO and LUMO are 7.05 eV and 7.07 eV, with the minimum HOMO/LUMO energy level difference being 0.02 eV (Figure 10a). These results indicate that both the monomer and dimer can be easily excited by UV light, which significantly facilitates the fast ISC process.

To theoretically understand the mechanisms underlying the enhanced ISC in MD molecules, we conducted TD-DFT calculations, including analysis of vertical excitation energies level and SOC. The TD-DFT results indicated that several triplet excited states (T_n_) were close in energy to the first singlet excited state (S_1_) with small ∆E_st_, thereby facilitating efficient ISC (Table 12 and Table 13). Furthermore, the S_1_ to T_n_ channels for ISC can be distinguished by their SOC constants, as determined through Dalton calculations (Figure 10b). The results revealed that UV-irradiated MD was excited from the ground state (S_0_) to S_1_, followed by ISC to the triplet states (T_1_) [49]. The S_1_ to T_1_ transition was accompanied by rapid vibrational relaxation processes that mediate spin multiplicity transitions from S = 1 to S = 3 states [50]. The dimer configuration exhibited a significantly reduced singlet–triplet energy gap (ΔE_st_ = 0.051 eV) compared with that of the monomer (ΔE_st_ = 0.111 eV). This narrow gap considerably enhanced the probability of ISC through the SOC process. The HOMO/LUMO energy level distribution results indicated that the S_1_ to T_1_ contributions of the monomer were greater than that of the dimer. These findings suggested that the monomer possessed a richer energy level with extended off-domains, which might facilitate enhanced phosphorescence. Notably, the SOC constants of MD were significantly larger than those of typical organic molecules [50]. This could be attributed to the smaller single heavy state–triple state gap (ΔE_ST_ < ±0.37 eV) and the higher SOC value (SOC > 0.3 cm^−1^) (Figure 10c,d). The number of more readily accessible ISC channels in dimers was five, whereas in monomers it was four. However, the SOC constants for the monomer at the smaller single state to triple excited state gaps were as follows: S_1_/T_1_: 0.811, S_1_/T_2_: 1.07, S_1_/T_3_: 4.97, S_1_/T_4_: 0.40, and S_1_/T_5_: 10.85. In comparison, the SOC constants for the dimer were S_1_/T_1_: 2.74, S_1_/T_2_: 5.51, S_1_/T_3_: 14.57, S_1_/T_4_: 11.22. In systems with small singlet–triplet energy gaps, monomeric species exhibited higher SOC constants (S_n_/T_n_) than dimers, which enhanced excitation probabilities. Although they exhibited the largest S_1_ to T_1_ energy separation, their strong SOC across multiple excited states facilitates ISC, resulting in significant triplet exciton generation.

**Figure 11:**
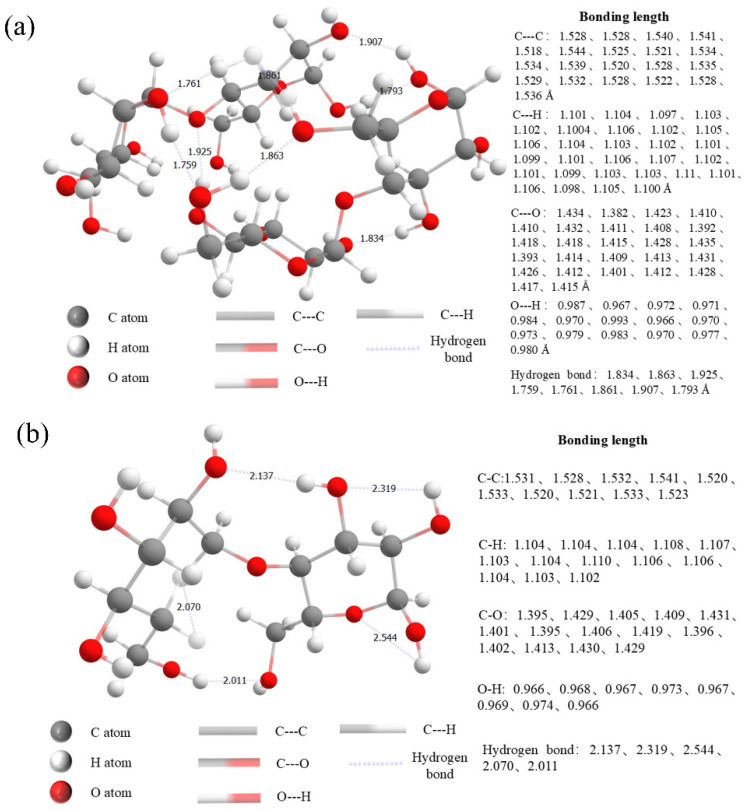
(**a**) MD dimer structure of the tablet with denoted intramolecular bonding interactions. (**b**) MD monomer structure of the tablet with denoted intramolecular bonding interactions.

Furthermore, structural optimization of MD monomers and dimers was performed using first-principles calculations with the ORCA quantum chemistry software, indicating a correlation between intramolecular conformation and phosphorescence excitation mechanisms. Intramolecular interaction also existed in the MD, specifically ···O···H, ···O···C, ···C···C, ···C···H interactions, as well as hydrogen bonds. The average bond lengths of these interactions in optimized structures were 0.969 Å for ···O···H, 1.105 Å for ···O···C, 1.528 Å for ···C···C, 1.411 Å for ···C···H, and 2.216 Å for hydrogen bonds (Figure 11a). In contrast, the average intramolecular bond lengths of the monomer were 0.976 Å, 1.103 Å, 1.530 Å, 1.414 Å and 1.838 Å, respectively (Figure 11b). Analysis of bond energy parameters of the optimized monomer and dimer structures showed that MD possessed a rigid structural environment, which was necessary for phosphorescence emission [25]. Besides hydrogen bonds, the contraction of interatomic distances in other chemical bonds within the monomer enhanced bond interactions. While this structure affected the intramolecular hydrogen bonding network, MDs with a high degree of hydrolysis facilitated the formation of a more extensive intermolecular hydrogen bonding network. Structural optimization induced a contraction in average bond lengths, which promoted the formation of π-conjugation networks through strengthened orbital overlap. This process also improved the CTE mechanism of MD. Moreover, the high degree of hydrolysis of MD led to enhanced intermolecular interactions and the formation of a more rigid environment, which promoted the SOC behavior. From the results of the SOC constants calculated by TD-DFT, the SOC constants of the monomer were consistently larger than those of the dimer at all gap energy levels. Therefore, MD DE-47 exhibited stronger triple excited state emission than MD DE-29. The RTP properties of MD increased with increasing DE values, which is a trend consistent with both the phosphorescence lifetime and the afterglow signal.

## 3. Materials and Methods

### 3.1. Materials

Maltodextrin GLUCIDEX-2 (MD DE-2, batch number 323205), maltodextrin GLUCIDEX-6 (MD DE-6, batch number 484807), maltodextrin GLUCIDEX-12 (MD DE-12, batch number 582134), maltodextrin GLUCIDEX-19 (MD DE-19, batch number 881497), dried glucose syrup GLUCIDEX-29 (MD DE-29, batch number 914998), dried glucose syrup GLUCIDEX-39 (MD DE-39, batch number 831761), and dried glucose syrup GLUCIDEX-47 (MD DE-47, batch number 268616-4) were supplied by Roquette Frères (Lille, France). Polyvinylpyrrolidone K30 (PVP K30, batch number 2450181) was purchased from Ashland Group Co., Ltd. (Wilmington, DE, USA). Glucose was provided by Shanghai Macklin Biochemical Technology (Shanghai, China) Co., Ltd. Potassium sulfate (K_2_SO_4_, batch number 2107231) and dicalcium phosphate (KCl, batch number Lot#C16777601) were purchased from Xilong Science Co., Ltd. (Guangzhou, China). Potassium carbonate (K_2_CO_3_, batch number 20210528) and magnesium stearate (MgSt, batch number 20240318) were from Sinopharm Chemical Reagent Co., Ltd. (Shanghai, China). Lithium chloride (LiCl, batch number Lot#C16453020) was purchased from Shanghai Macklin Biochemical Technology Co., Ltd. (Shanghai, China). Vaseline was purchased from Shandong Anjie Gaoke Disinfection Technology Co., Ltd. (Dezhou, China).

### 3.2. Preparation of Tablets

The samples were directly compressed into tablets using an ERWEKA-TRD8 multifunctional tablet press (Langen, Germany) with a 10 mm round flat punch. The compaction speed was 4.2 mm/s. and the prepared tablet hardnesses were controlled at 100 N. The tablet’s weight was about 300 mg [51].

### 3.3. Afterglow Photography

The afterglow photographs of every tablet were captured using a camera. A UV light at 254 nm was fixed at a distance of 5 cm from the surface of the tablets, and the afterglow photographs were recorded for 5 s after irradiation with the UV light radiation for 10 s [34]. The experiment was conducted in a dark natural environment.

Each tablet’s afterglow photograph was captured and analyzed frame by frame using the video analysis software ‘Cupcat’ (version 5.7.0). The luminance of the afterglow photographs was quantified using the ‘ImageJ’ (version 1.5.3) software, which analyzed the average gray value of afterglow photographs [52].

### 3.4. Phosphorescence Spectra and Phosphorescence Lifetimes

The phosphorescence spectra and phosphorescence lifetime of each tablet were determined by a Edinburgh FLS1000 transient/steady state fluorescence spectrometer (Edinburgh, MN, USA). The device is equipped with a xenon arc lamp (Xe2), a microsecond flash lamp (μF900) and a nanosecond flash lamp (nF900) [53,54]. Each datapoint was collected by integrating total emission from a single flash with 0.1 ms gate time and 2 to 4 s total decay time. The phosphorescence lifetime decay curve was fitted by applying the multiexponential function Equation (1). The average phosphorescence lifetimes were calculated according to Equation (2) [27]. All tests were performed under natural environmental conditions.

(1) $$R\left(t\right)=\sum B_{i}\exp\left(-\frac{t}{\tau_{i}}\right)$$

(2) $$\tau_{avg}=\sum B_{i}\tau_{i}$$

where *R*(*t*) is phosphorescence lifetime, *B_i_* is the percentage of different types of phosphorescence lifetime, *t* is time, *τ_i_* is different types of phosphorescence lifetime, and *τ_avg_* is average phosphorescence lifetime.

### 3.5. Fourier Transform Infrared Spectroscopy (FTIR)

Potassium bromide and MD powder were fully mixed at a mass ratio of 100:1, and the sample powder was compacted using a HY-15 tablet press (Tianjin, China). It was placed into a preheated FTIR spectrometer for analyzing the chemical structures [55].

### 3.6. Pressure Response

Tablets of various hardness levels with 70, 100, 130, and 160 N were prepared using the ERWEKA-TRD8 multifunctional tablet press using a 10 mm round flat punch. The tablet weight was 300 mg [56]. All tablet samples were dried at 60 °C for 6 h before afterglow imaging, removing the influence of moisture. The afterglow photographs of samples were taken following the method detailed in Section 3.3.

### 3.7. Hygroscopicity

The MD tablets with 100 N were used for hygroscopicity testing. To maintain designated RH levels, the tablets were stored in different closed containers at 25 °C. Every container was filled with a different saturated salt solution, i.e., potassium sulfate (K_2_SO_4_), potassium chloride (KCl), potassium carbonate (K_2_CO_3_), and lithium chloride (LiCl), establishing RH environments of 94%, 84%, 43%, and 11%, respectively [17,57]. Initially, a saturated salt solution was placed at the bottom of the container. A dried weighing bottle was positioned on the container platform for 24 h to ensure equilibrium of environmental conditions. Subsequently, the initial weight of the weighing bottle (*m*_0_) was recorded. The tablet was placed into the weighing bottle and its combined weight (*m*_1_) was measured. Thereafter, the weighing bottle was weighed (*m*_2_) every 1 h. The moisture content was calculated as shown in Equation (3):

(3) $$moisture\;content=\frac{m_{2}-m_{1}}{m_{1}-m_{0}}*100\%$$

The afterglow photographs and phosphorescence lifetimes of all samples were measured in accordance with the method in Section 3.3 and Section 3.4, respectively.

### 3.8. Temperature Response

The tablets with 100 N were used for temperature response testing. The samples were subjected to a series of controlled exposures at three temperatures, i.e., 0 °C, 25 °C, and 40 °C. The afterglow photographs were taken and variable-temperature phosphorescence lifetimes of all samples were measured with the methods in Section 3.3 and Section 3.4, respectively.

### 3.9. Oxygen Response

All tablets with 100 N were dried at 60 °C for 6 h before oxygen response testing, removing the influence of moisture. Tablets were stored in different conditions for 6 h at 25 °C, i.e., ambient air contact and vacuum drying. The afterglow photographs were taken and phosphorescence lifetimes of all samples were measured in accordance with the method in Section 3.3 and Section 3.4, respectively.

### 3.10. Photostability

The tablets with 100 N were used for photostability testing. Prolonged UV irradiation at the optimal excitation wavelength was used to measure the afterglow decay of the MD tablets every hour. The afterglow photographs were taken and phosphorescence lifetimes of all samples were measured in accordance with the method in Section 3.3 and Section 3.4, respectively.

### 3.11. Application

#### 3.11.1. Tablets with Non-Afterglow Effect Materials

Tablets were prepared by mixing MD and non-afterglow-effect PVP K30 at various mixing ratios (2:8, 4:6, 6:4, 8:2). The tablet weight was 300 mg, and the hardness of the tablet was 100 N. The afterglow photographs of all samples were taken after heating at 60 °C for 6 h, which was performed in accordance with the method in Section 3.3.

#### 3.11.2. Uniformity and Non-Uniformity of Tablets

The uniformity and non-uniformity of tablets were evaluated by analyzing the afterglow photographs of tablets. Uniform tablets were prepared by physically shaking materials and non-uniform tablets with inhomogeneous content were not shaken. Parameters of tablets were used to assess the afterglow photographs, such as the gray value and RSD. The afterglow photographs of all tablets were characterized according to the method in Section 3.3.

### 3.12. Theoretical Calculation

#### 3.12.1. TD-DFT Calculation

All-electron density functional theory (DFT) computations were performed using ORCA quantum chemistry software (version 6.0.0). The hybrid B3LYP functional was employed for all calculations. Geometry optimization utilized the def2-SVP basis set, augmented by the DFT-D3 empirical dispersion correction to account for weak intermolecular interaction to improve the calculation accuracy. Subsequent excited state analyses and SOC calculations employed the def2-TZVP basis set while maintaining the B3LYP functional framework. SOC values were computed through the spin–orbit mean-field method [25].

#### 3.12.2. HOMO and LUMO Electron Density Calculations

The HOMO and LUMO energy densities were obtained with GaussView 5.0.8 (isovalue—0.02, cubic mesh—coarse) [34].

## 4. Conclusions

In summary, we have explored the stimuli-responsive properties of MD with different DE values. As a non-conventional luminescent stimuli-responsive RTP material, the phosphorescence emission of MD originates from the hydrogen bonding networks formed by hydroxyl and carbonyl groups. MD tablets with different DE values exhibited RTP emission with phosphorescence lifetimes of 186.91~618.85 ms and afterglow imaging with 1~4 s. A higher DE value corresponded to a stronger phosphorescence lifetime and afterglow effect. However, the afterglow effect of MD tablets was less affected by the hardness. The moisture content of MD tablets weakened the rigid environment by disrupting intramolecular hydrogen bonding networks. Lowering the temperature restricted molecular motions, including the vibration and rotation of the hydroxyl bonds in the MD tablet. Furthermore, oxygen and UV light acted as strong phosphorescence quenchers, inhibiting the phosphorescence emission. MD DE-39 and DE-37 tablets demonstrated excellent resistance to prolonged UV light exposure and ambient air. In addition, this non-destructive detection exhibited a lower signal-to-background ratio compared with the traditional fluorescence detection method. The uniformity of tablets can be non-destructively detected by analyzing the RSD values of the afterglow photographs of MD tablets. In a word, the afterglow signal of tablets can be used to determine the quality of tablets affected by environmental factors, e.g., RH, temperature, oxygen, UV light, etc., during transportation and storage, thereby reducing medicinal risks. Currently, rapid, simple and effective non-destructive detection methods are increasingly being adopted. The new detection method enables the quality inspection of tablets to shift from offline to online detection in real time.

## Figures and Tables

**Table 1:** Phosphorescence lifetimes (*τ*_ave_) and afterglow frames of MD tablets with different DE values and glucose.

Sample	*τ*_1_ (ms)	*B*_1_ (%)	*τ*_2_ (ms)	*B*_2_ (%)	*τ*_3_ (ms)	*B*_3_ (%)	*τ*_ave_ (ms)	Afterglow Frame (Frame)
DE-2	13.79	9.09	89.74	37.49	333.35	53.42	212.97	105.3 ± 8.3 ^c^
DE-6	9.03	12.97	90.81	42.12	328.42	44.91	186.91	28.6 ± 3.0 ^f^
DE-12	6.65	4.22	67.74	29.66	355.54	66.12	255.46	52.0 ± 4.0 ^e^
DE-19	11.91	7.21	89.40	27.90	430.34	64.89	305.05	80.0 ± 7.2 ^d^
DE-29	9.42	7.64	93.09	21.55	561.71	70.81	418.53	106.0 ± 5.2 ^c^
DE-39	15.77	6.22	149.51	25.17	767.99	68.61	565.53	170.0 ± 3.4 ^b^
DE-47	22.86	6.58	199.60	24.73	826.76	68.70	618.85	271.3 ± 1.1 ^a^
Glucose	14.68	45.80	85.10	54.20	-	-	52.84	0

The sample represents all tablet samples. Determined from the fitting function of $R\left(t\right)=\sum B_{i}\exp\left(-\frac{t}{\tau_{i}}\right)$ and $\tau_{avg}=\sum B_{i}\tau_{i}$ according to the decay curves, where *R*(*t*) is phosphorescence lifetime, *t* is time. *B*_1_, *B*_2_, *B*_3_ are the percentages of different types of phosphorescence lifetime, *τ*_1_, *τ*_2_, *τ*_3_ are different types of phosphorescence lifetime, and *τ_avg_* is average phosphorescence lifetime. Afterglow time (s) = (afterglow frame/60). ANOVA (a–f), *p* < 0.05 (Same letters indicate no significant difference between groups, different letters indicate significant difference between groups. The more letters separating between the two groups, the more significant the difference between the groups).

**Table 2:** Afterglow frames for MD tablet afterglow photographs with different DE values and different hardnesses ($\bar{x}$ ± SD, *n* = 3).

Sample	Afterglow Frame (Frame)			
	70 N	100 N	130 N	160 N
DE-2	108.0 ± 4.0	105.3 ± 8.3	106.6 ± 6.4	108.0 ± 4.0
DE-6	27.3 ± 5.0	28.6 ± 3.0	34.6 ± 2.3	30.0 ± 6.9
DE-12	51.3 ± 5.0	52.0 ± 4.0	54.6 ± 2.3	49.3 ± 2.3
DE-19	83.3 ± 1.1	80.0 ± 7.2	102.6 ± 1.1	98.0 ± 10.0
DE-29	102.6 ± 1.1	106.0 ± 5.2	94.6 ± 7.0	96.0 ± 5.2
DE-39	178.0 ± 14.0	170.0 ± 3.4	176.6 ± 5.0	182.6 ± 1.1
DE-47	254.0 ± 2.0	271.3 ± 1.1	258.6 ± 5.7	268.6 ± 5.7

Afterglow time (s) = (afterglow frame/60).

**Table 3:** Phosphorescence lifetimes (*τ*_ave_) and moisture content of MD tablets with different DE values stored in different RH levels for hygroscopicity testing for 6 h.

Sample	RH (%)	Moisture Content (%)	*τ*_1_ (ms)	*B*_1_ (%)	*τ*_2_ (ms)	*B*_2_ (%)	*τ*_3_ (ms)	*B*_3_ (%)	*τ*_ave_ (ms)
DE-2	94	11.39 ± 0.17	7.81	18.27	30.93	52.29	131.17	29.44	56.21
	83	9.41 ± 0.11	4.92	9.02	37.02	51.09	135.52	39.89	73.41
	43	6.84 ± 0.59	17.87	12.54	87.99	42.53	276.81	44.93	164.03
	11	5.37 ± 0.18	15.68	8.53	98.60	39.33	337.09	52.14	215.87
DE-6	94	10.98 ± 0.26	14.63	44.22	70.06	45.44	183.48	10.23	57.07
	83	9.40 ± 0.16	8.30	21.82	43.90	50.79	147.80	27.39	64.59
	43	4.78 ± 0.48	11.02	15.92	69.41	37.87	256.01	46.85	147.98
	11	2.51 ± 0.40	8.18	10.27	67.99	33.29	321.86	56.44	205.13
DE-12	94	10.69 ± 0.13	12.22	12.39	67.56	53.04	214.77	34.57	111.60
	83	9.03 ± 0.14	18.59	17.86	108.49	49.90	300.60	32.23	154.34
	43	5.77 ± 0.23	10.97	6.83	90.44	31.47	359.99	61.70	251.33
	11	2.37 ± 0.10	10.92	5.90	102.18	30.09	452.35	64.02	320.98
DE-19	94	9.84 ± 0.48	21.07	14.71	118.60	40.68	364.39	44.62	213.94
	83	7.11 ± 0.11	12.80	8.85	87.43	31.75	351.23	59.40	237.52
	43	4.22 ± 0.24	20.29	10.46	153.02	28.65	577.86	60.89	397.82
	11	1.92 ± 0.09	16.25	8.57	132.21	29.97	608.75	61.46	415.15
DE-29	94	7.84 ± 0.06	10.74	6.80	85.38	23.70	520.34	69.50	382.61
	83	6.49 ± 0.10	7.24	6.41	85.74	22.43	518.18	71.16	388.43
	43	3.29 ± 0.16	13.63	9.66	131.23	27.92	577.15	62.42	398.22
	11	1.07 ± 0.02	9.86	7.84	105.26	26.30	593.70	65.86	419.47
DE-39	94	6.40 ± 0.31	19.66	6.04	154.49	27.60	776.62	65.36	551.43
	83	4.54 ± 0.58	21.17	6.77	160.57	28.81	797.16	64.43	561.31
	43	2.11 ± 0.19	23.19	6.71	165.07	27.77	795.47	65.52	568.59
	11	0.99 ± 0.02	21.83	6.58	174.89	28.08	795.20	65.33	570.05
DE-47	94	6.35 ± 0.08	24.75	7.81	166.34	30.82	776.76	61.37	529.89
	83	4.56 ± 0.32	14.83	4.27	151.53	23.84	758.40	71.89	581.97
	43	3.52 ± 0.22	23.86	6.08	184.24	23.66	782.52	70.26	594.84
	11	1.48 ± 0.04	20.45	5.24	175.27	23.54	784.47	71.22	601.03

Determined from the fitting function of $R\left(t\right)=\sum B_{i}\exp\left(-\frac{t}{\tau_{i}}\right)$ and $\tau_{avg}=\sum B_{i}\tau_{i}$ according to the decay curves, where *R*(*t*) is phosphorescence lifetime, *t* is time. *B*_1_, *B*_2_, *B*_3_ are the percentages of different types of phosphorescence lifetime, *τ*_1_, *τ*_2_, *τ*_3_ are different types of phosphorescence lifetime, and *τ_avg_* is average phosphorescence lifetime.

**Table 4:** Afterglow frames and initial gray value of MD tablets with different DE values stored at different RH levels ($\bar{x}$ ± SD, *n* = 3).

Sample	Afterglow Frame (Frame)				Initial Gray Value			
	RH 94%	RH 83%	RH 43%	RH 11%	RH 94%	RH 83%	RH 43%	RH 11%
DE-2	3.3 ± 1.1	11.3 ± 2.3	20.6 ± 2.3	42.6 ± 1.1	0.293 ± 0.107	0.845 ± 0.102	1.201 ± 0.085	1.740 ± 0.074
DE-6	4.6 ± 3.0	7.3 ± 1.1	21.3 ± 1.1	41.3 ± 1.1	0.363 ± 0.226	0.514 ± 0.210	1.201 ± 0.169	1.844 ± 0.063
DE-12	8.0 ± 2.0	9.3 ± 1.1	25.3 ± 2.3	44.6 ± 3.0	0.529 ± 0.213	0.650 ± 0.156	1.340 ± 0.114	1.664 ± 0.131
DE-19	15.3 ± 1.1	19.3 ± 1.1	67.3 ± 5.0	83.3 ± 4.6	1.082 ± 0.194	1.753 ± 0.194	1.920 ± 0.197	2.085 ± 0.068
DE-29	14.6 ± 3.0	54.0 ± 3.4	95.3 ± 9.4	96.6 ± 3.0	0.595 ± 0.103	1.172 ± 0.278	1.719 ± 0.160	1.645 ± 0.273
DE-39	110.0 ± 10.5	170.0 ± 2.0	190.6 ± 6.1	194.0 ± 2.0	1.445 ± 0.242	2.081 ± 0.090	2.325 ± 0.036	2.421 ± 0.062
DE-47	109.3 ± 9.8	124.0 ± 17	189.3 ± 4.6	204.0 ± 7.2	1.145 ± 0.049	1.574 ± 0.098	2.253 ± 0.062	2.499 ± 0.121

Afterglow time (s) = (afterglow frame/60).

**Table 5:** Afterglow frames and initial gray values of MD tablets with different DE values under different temperatures ($\bar{x}$ ± SD, *n* = 3).

Sample	Afterglow Frame (Frame)			Initial Gray Value		
	0 °C	25 °C	40 °C	0 °C	25 °C	40 °C
DE-2	98.0 ± 6.0	77.3 ± 5.0	73.3 ± 5.0	2.152 ± 0.033	1.763 ± 0.039	1.949 ± 0.045
DE-6	63.3 ± 1.1	54.6 ± 5.7	50.0 ± 5.2	1.556 ± 0.231	1.442 ± 0.064	1.583 ± 0.100
DE-12	88.6 ± 9.4	80.0 ± 5.2	58.6 ± 1.1	1.642 ± 0.172	1.543 ± 0.047	1.533 ± 0.383
DE-19	136.6 ± 9.0	115.0 ± 7.0	95.3 ± 1.1	1.797 ± 0.189	1.657 ± 0.126	1.755 ± 0.170
DE-29	140.0 ± 13.1	108.6 ± 5.7	102.0 ± 0.0	1.887 ± 0.102	1.681 ± 0.198	1.785 ± 0.105
DE-39	239.3 ± 4.6	202.6 ± 8.3	181.3 ± 3.0	2.132 ± 0.024	2.280 ± 0.259	2.197 ± 0.398
DE-47	260.6 ± 9.8	239.3 ± 4.6	211.3 ± 3.0	2.208 ± 0.088	2.247 ± 0.165	2.175 ± 0.038

Afterglow time (s) = (afterglow frame/60).

**Table 6:** Phosphorescence lifetimes (*τ*_ave_) of MD DE-2 tablets stored at different temperatures.

Sample	Temperature (K)	*τ*_1_ (ms)	*B*_1_ (%)	*τ*_2_ (ms)	*B*_2_ (%)	*τ*_ave_ (ms)
DE-2	273	53.65	19.35	445.03	80.65	369.30
DE-2	298	29.82	23.25	258.98	76.75	205.70
DE-2	313	24.10	29.53	177.01	70.47	131.86

Determined from the fitting function of $R\left(t\right)=\sum B_{i}\exp\left(-\frac{t}{\tau_{i}}\right)$ and $\tau_{avg}=\sum B_{i}\tau_{i}$ according to the decay curves, where *R*(*t*) is phosphorescence lifetime, *t* is time. *B*_1_, *B*_2_, *B*_3_ are the percentages of different types of phosphorescence lifetime, *τ*_1_, *τ*_2_, *τ*_3_ are different types of phosphorescence lifetime, and *τ_avg_* is average phosphorescence lifetime.

**Table 7:** Phosphorescence lifetimes (*τ*_ave_) and afterglow frames of MD tablets with different DE values under UV radiation of 3 h.

Sample	*τ*_1_ (ms)	*B*_1_ (%)	*τ*_2_ (ms)	*B*_2_ (%)	*τ*_3_ (ms)	*B*_3_ (%)	*τ*_ave_ (ms)	Afterglow Frame (Frame)
DE-2	7.54	16.33	58.44	44.76	195.68	38.91	103.53	28.0 ± 2.0
DE-6	8.85	15.14	60.15	53.46	244.46	31.40	110.26	9.3 ± 2.3
DE-12	21.32	20.60	161.89	50.97	390.06	28.44	197.84	16.0 ± 2.0
DE-19	16.29	8.06	118.45	27.80	562.35	64.14	225.04	64.0 ± 7.2
DE-29	7.34	12.41	77.63	35.70	512.12	51.98	294.82	74.0 ± 7.2
DE-39	16.63	7.11	128.76	25.29	754.35	67.60	543.68	163.3 ± 8.0
DE-47	14.46	5.62	123.66	24.15	752.62	70.23	559.24	220.0 ± 4.0

Determined from the fitting function of $R\left(t\right)=\sum B_{i}\exp\left(-\frac{t}{\tau_{i}}\right)$ and $\tau_{avg}=\sum B_{i}\tau_{i}$ according to the decay curves, where *R*(*t*) is phosphorescence lifetime, *t* is time. *B*_1_, *B*_2_, *B*_3_ are the percentages of different types of phosphorescence lifetime, *τ*_1_, *τ*_2_, *τ*_3_ are different types of phosphorescence lifetime, and *τ_avg_* is average phosphorescence lifetime. Afterglow time (s) = (afterglow frame/60).

**Table 8:** Afterglow frames for MD tablets with different DE values under vacuum drying and ambient air contact conditions ($\bar{x}$ ± SD, *n* = 3).

Sample	Afterglow Frame (Frame)	
	Vacuum Drying	Ambient Air Contact
DE-2	65.3 ± 2.3	34.6 ± 5.7
DE-6	40.0 ± 3.4	22.0 ± 2.0
DE-12	82.0 ± 0.0	50.6 ± 4.1
DE-19	103.3 ± 2.3	96.0 ± 0.0
DE-29	116.0 ± 0.0	89.3 ± 2.3
DE-39	202.0 ± 2.0	188.0 ± 3.4
DE-47	245.3 ± 5.7	238.6 ± 5.7

Afterglow time (s) = (afterglow frame/60).

**Table 9:** Phosphorescence lifetimes (*τ*_ave_) of MD tablets with different DE values under vacuum-drying environments.

Sample	*τ*_1_ (ms)	*B*_1_ (%)	*τ*_2_ (ms)	*B*_2_ (%)	*τ*_3_ (ms)	*B*_3_ (%)	*τ*_ave_ (ms)
DE-2	12.35	6.92	80.75	35.33	362.09	57.75	238.49
DE-6	14.23	14.81	103.92	36.47	365.86	48.71	218.21
DE-12	12.57	6.30	92.97	28.32	410.55	65.38	295.54
DE-19	16.70	9.10	122.90	31.72	576.86	59.18	381.89
DE-29	18.12	10.02	149.78	27.08	778.30	62.89	531.85
DE-39	16.75	5.66	158.64	26.50	803.40	67.84	588.02
DE-47	20.38	4.97	183.75	24.38	820.18	70.65	625.27

Determined from the fitting function of $R\left(t\right)=\sum B_{i}\exp\left(-\frac{t}{\tau_{i}}\right)$ and $\tau_{avg}=\sum B_{i}\tau_{i}$ according to the decay curves, where *R*(*t*) is phosphorescence lifetime, *t* is time. *B*_1_, *B*_2_, *B*_3_ are the percentages of different types of phosphorescence lifetime, *τ*_1_, *τ*_2_, *τ*_3_ are different types of phosphorescence lifetime, and *τ_avg_* is average phosphorescence lifetime. Afterglow time (s) = (afterglow frame/60).

**Table 10:** Relative standard deviation (RSD) of the afterglow photographs of uniform MD tablets with different DE values.

Sample	RSD (%)		
	1	2	3
DE-2	7.21	6.37	7.19
DE-6	19.80	10.80	8.27
DE-12	13.09	15.00	8.37
DE-19	12.76	14.38	5.52
DE-29	7.08	6.60	8.43
DE-39	4.97	3.91	5.38
DE-47	8.34	6.13	6.39

**Table 11:** RSD of the afterglow photographs of non-uniform MD tablets with different DE values.

Sample	RSD (%)		
	1	2	3
DE-2	42.00	35.79	22.87
DE-6	45.47	64.77	70.05
DE-12	72.40	21.16	131.03
DE-19	68.44	68.37	69.95
DE-29	35.52	63.72	31.35
DE-39	75.36	82.10	92.95
DE-47	29.48	55.39	90.53

**Table 12:** TD-DFT-calculated singlet and triplet excited states transition configurations of dimer (DE-47).

Excited State	n-th	Energy (eV)	Transition Configuration (%)
S_n_	1	6.4070	H→L (86.8%), H-1→L (7.3%), H-2→L (1.3%)
T_n_	1	6.3560	H→L (81.9%), H-1→L(7.0%), H-2→L(2.1%), H-4→L(1.3%)
	2	6.5690	H-1→L (77.3%), H→L (6.3%), H-1→L + 3 (4.6%), H-3→L (3.5%), H-1→L + 2 (1.7%), H-2→L (1.0%)
	3	6.6350	H-2→L (34.4%), H-3→L (31.7%), H-1→L (4.0%), H-5→L (3.4%), H-4→L (3.2%), H-6→L (2.9%), H-7→L (2.5%), H-9→L (2.3%), H-3→L + 3 (1.5%), H→L (1.4%)
	4	6.6860	H-1→L + 1 (79.8%), H→L + 1 (9.3%), H-1→L + 3 (1.7%), H-11→L + 1 (1.5%), H-4→L + 1 (1.4%)
	5	6.7020	H-2→L (32.5%), H-3→L (20.3%), H-8→L (10.4%), H-6→L (5.8%), H-7→L (5.7%), H-19→L (3.5%), H-5→L (2.2%), H-1→L + 1 (1.4%), H-16→L (1.2%), H→L (1.2%), H-22→L (1.2%), H→L + 1 (1.2%), H-1→L (1.1%), H-6→L + 1 (1.0%)
	6	6.7900	H-4→L (25.4%), H-5→L (10.5%), H-2→L (9.7%), H-3→L (7.8%), H-8→L (6.9%), H-6→L (5.3%), H-7→L (4.7%), H-19→L (3.9%), H→L (3.3%), H-16→L (1.9%), H→L + 1 (1.8%), H-22→L (1.6%), H-10→L (1.3%), H-6→L + 1 (1.2%), H-21→L (1.1%)
	7	6.8230	H→L + 1 (49.2%), H-6→L + 1 (7.9%), H-2→L + 1 (7.1%), H-1→L + 1 (6.7%), H-3→L + 1 (5.6%), H-2→L (3.5%), H-5→L + 1 (3.3%), H→L + 5 (2.7%), H-7→L + 1 (2.5%), H-8→L (1.3%)
	8	6.8890	H→L + 1 (24.4%), H-6→L + 1 (12.4%), H-5→L + 1 (11.4%), H-4→L + 1 (9.6%), H-3→L + 1 (9.3%), H-8→L (3.8%), H-2→L + 1 (3.1%), H-7→L + 1 (2.7%), H-19→L (2.4%), H-1→L + 1 (1.4%), H→L + 5 (1.4%)
	9	6.9150	H-2→L + 1 (30.5%), H-6→L + 1 (8.4%), H-2→L + 5 (5.5%), H-4→L + 1 (3.4%), H-8→L + 1 (3.1%), H-3→L + 1 (3.0%), H-2→L + 3 (3.0%), H-2→L + 7 (2.5%), H-7→L + 1 (2.1%), H-2→L + 4 (1.3%), H-2→L + 2 (2.0%), H-4→L + 3 (1.4%), H-4→L + 5 (1.3%), H-11→L + 1 (1.3%), H-12→L + 1 (1.3%), H-6→L (1.3%), H-15→L + 1 (1.2%), H-2→L + 6 (1.2%), H-6→L + 5 (1.2%), H-6→L + 7 (1.1%)
	10	6.9240	H-1→L + 3 (28.2%), H-1→L + 2(15.3%), H-1→L (7.4%), H-1→L + 4 (6.7%), H-1→L + 8 (6.1%), H-1→L + 7 (5.9%), H-1→L + 9 (4.5%), H-1→L + 5 (3.7%), H-1→L + 10 (2.6%), H→L + 3 (2.6%), H-1→L + 12 (2.2%), H→L + 2 (1.5%)

**Table 13:** TD-DFT-calculated singlet and triplet excited states transition configurations of monomer (DE-29).

Excited State	n-th	Energy (eV)	Transition Configuration (%)
Sn	1	6.3940	H→L (63.8%), H-2→L (29.0%), H-1→L (2.3%), H-5→L (1.6%), H-8→L (1.1%)
Tn	1	6.2830	H→L (81.9%), H-1→L(7.0%), H-2→L(2.1%), H-4→L(1.3%)
	2	6.5090	H→L + 1 (62.0%), H→L + 4 (4.7%), H→L (4.1%), H-2→L + 1 (3.7%), H-1→L (3.5%), H-1→L + 1 (3.3%), H-6→L + 1 (3.1%), H→L + 2 (1.2%), H-8→L + 1 (1.1%), H→L + 8 (1.1%)
	3	6.5190	H-1→L (64.8%), H→L (13.5%), H→L + 1 (5.4%), H-4→L (4.2%), H-3→L (3.1%), H-1→L + 5 (1.5%), H-6→L (1.2%)
	4	6.6270	H-2→L (39.7%), H→L (34.5%), H-1→L (11.9%), H-5→L (4.7%), H→L + 2 (2.1%), H-8→L (1.5%)
	5	6.8020	H→L + 2 (57.7%), H-6→L + 2 (11.8%), H-4→L + 2 (8.6%), H-2→L + 2 (3.1%), H→L + 1 (2.4%), H-7→L + 2 (1.9%), H→L + 9 (1.5%), H→L (1.4%), H-8→L + 2 (1.1%)
	6	6.8460	H-3→L (50.2%), H-5→L (16.8%), H-4→L (6.4%), H-1→L (3.6%), H-2→L (3.3%), H-3→L + 5 (2.8%), H-8→L (2.3%), H-2→L + 1 (1.5%), H-5→L + 1 (1.4%), H-5→L + 5, (1.1%)
	7	6.8930	H-5→L (14.8%), H-4→L + 2 (14.6%), H-3→L (13.3%), H-2→L + 1 (9.3%), H-2→L + 2 (8.4%), H-6→L + 2 (6.6%), H→L + 2 (3.3%), H-3→L + 2 (3.1%), H-8→L + 2 (2.7%), H-5→L + 1 (1.5%), H-3→L + 5 (1.5%), H-5→L + 2 (1.3%), H-8→L (1.3%), H-1→L (1.1%)
	8	6.9140	H-5→L (19.9%), H-4→L + 2 (11.7%), H-2→L + 2 (10.7%), H-3→L (8.0%), H-6→L + 2 (5.8%), H-8→L (5.4%), H→L + 2 (5.3%), H-5→L + 1 (3.7%), H-3→L + 2 (2.9%), H-8→L + 2 (2.1%), H-1→L (2.0%), H-6→L (1.6%), H-2→L + 1 (1.5%), H-3→L + 5 (1.4%), H-2→L (1.1%), H-2→L + 9 (1.0%)
	9	7.0280	H-2→L + 1 (36.4%), H-1→L + 1 (18.9%), H-5→L (8.8%), H-6→L (4.4%), H→L + 1 (3.5%), H-8→L (3.4%), H-3→L + 1 (3.2%), H-4→L (2.9%), H-2→L + 2 (2.7%), H-1→L (1.6%), H→L + 2 (1.5%), H-1→L + 4 (1.1%), H-5→L + 1 (1.0%)
	10	7.0520	H-2→L + 1 (22.1%), H-1→L + 1 (17.7%), H-4→L + 4 (13.4%), H-5→L (9.2%), H-6→L (6.9%), H-1→L + 5 (3.5%), H-3→L (3.3%), H-5→L + 1 (3.2%), H→L + 4 (3.0%), H-1→L + 3 (1.9%), H-1→L + 4 (1.8%), H-1→L (1.4%), H-2→L (1.4%)

## Data Availability

The original contributions presented in this study are included in the article. Further inquiries can be directed to the corresponding authors.

## Supplementary Materials

The following supporting information can be downloaded at: https://www.mdpi.com/article/10.3390/ph19040565/s1, Figure S1: Afterglow photographs of MD tablets with different DE values stored in UV irradiation environment for 1 h; Figure S2: Afterglow photographs of MD tablets with different DE values stored in UV irradiation environment for 2 h; Figure S3: Afterglow photographs of MD tablets with different DE values stored in UV irradiation environment for 3 h.

## Abbreviations

The following abbreviations are used in this manuscript:

API	Active pharmaceutical ingredient
CTE	Clustering-triggered emission
DE	Dextrose equivalents
ISC	Intersystem crossing
MD	Maltodextrin
PVP K30	Polyvinylpyrrolidone K30
RH	Relative humidity
RSD	Relative standard deviation
RTP	Room-temperature phosphorescence
SD	Standard deviation
SOC	Spin–orbit coupling
TD-DFT	Time-dependent density functional theory
UV	Ultraviolet
