# Supplementary material for: Edible Ultralong Organic Phosphorescent Maltodextrin with Different Dextrose Equivalents Values for Afterglow Visualizing the Quality of Tablets
Source: Pharmaceuticals (Basel). 2026 Apr 1;19(4):565. doi: 10.3390/ph19040565 (PMC13118293; doi:10.3390/ph19040565)
Supplement: Supplementary file 1 [file pharmaceuticals-19-00565-s001.zip › pharmaceuticals-4203279-supplementary.pdf]

Supporting information

Figure S1. Afterglow photographs of MD tablet with different DE value stored in UV irradiation environment for 1 h.

Figure S2. Afterglow photographs of MD tablet with different DE value stored in UV irradiation environment for 2 h.

Figure S3. Afterglow photographs of MD tablet with different DE value stored in UV irradiation environment for 3 h.

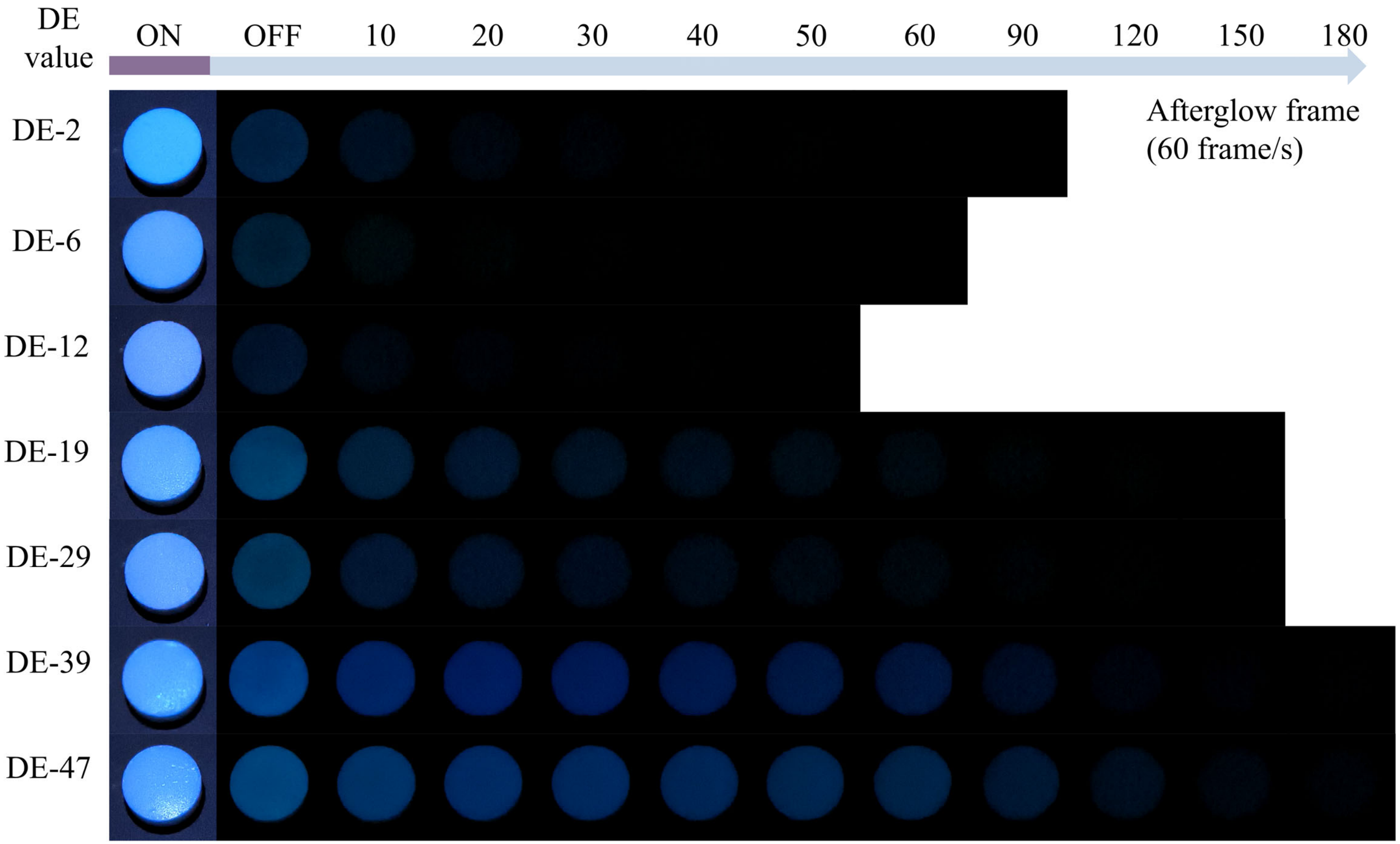

Figure S1. Afterglow photographs of MD tablet with different DE value stored in UV irradiation environment for 1 h.

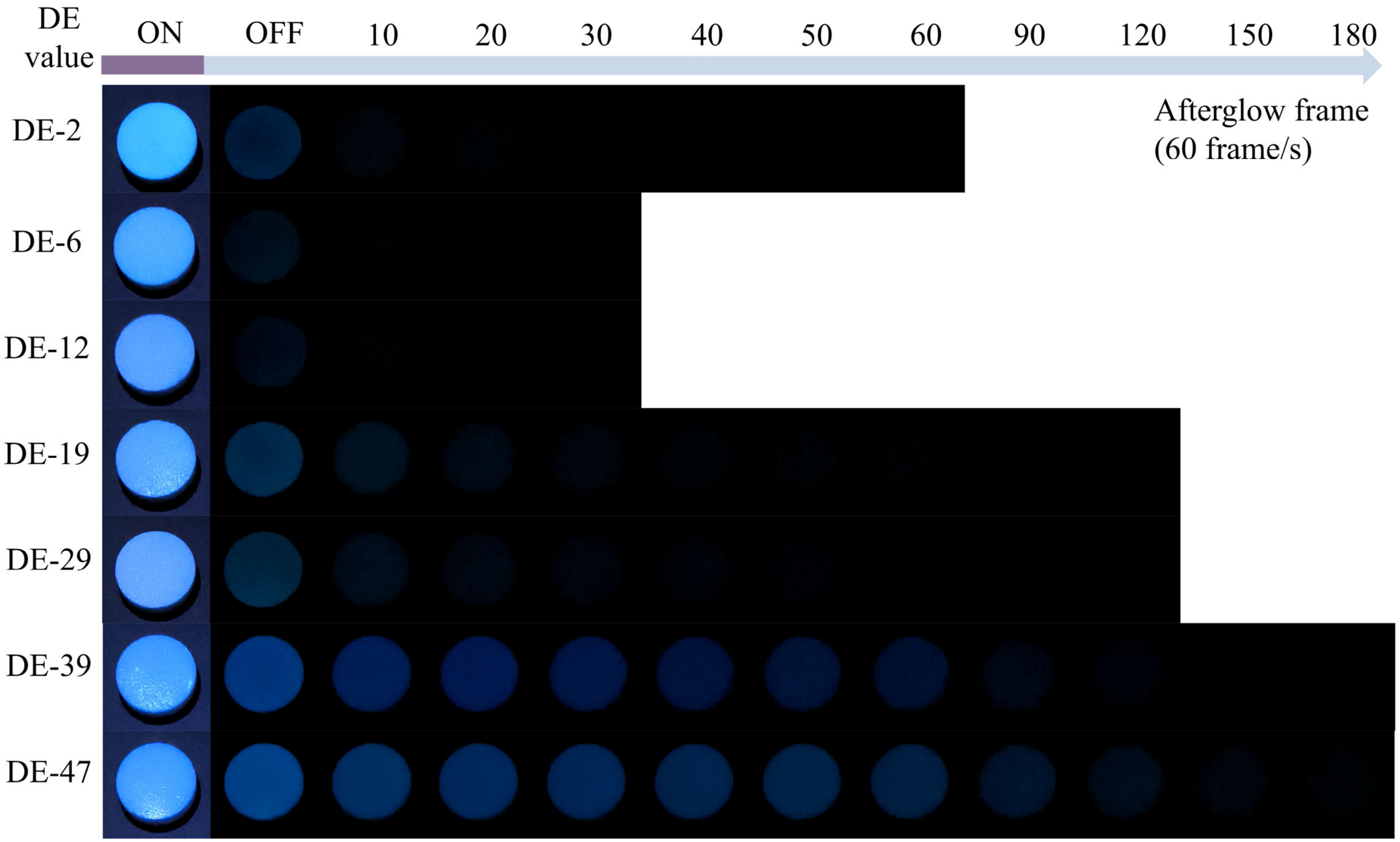

Figure S2. Afterglow photographs of MD tablet with different DE value stored in UV irradiation environment for 2 h.

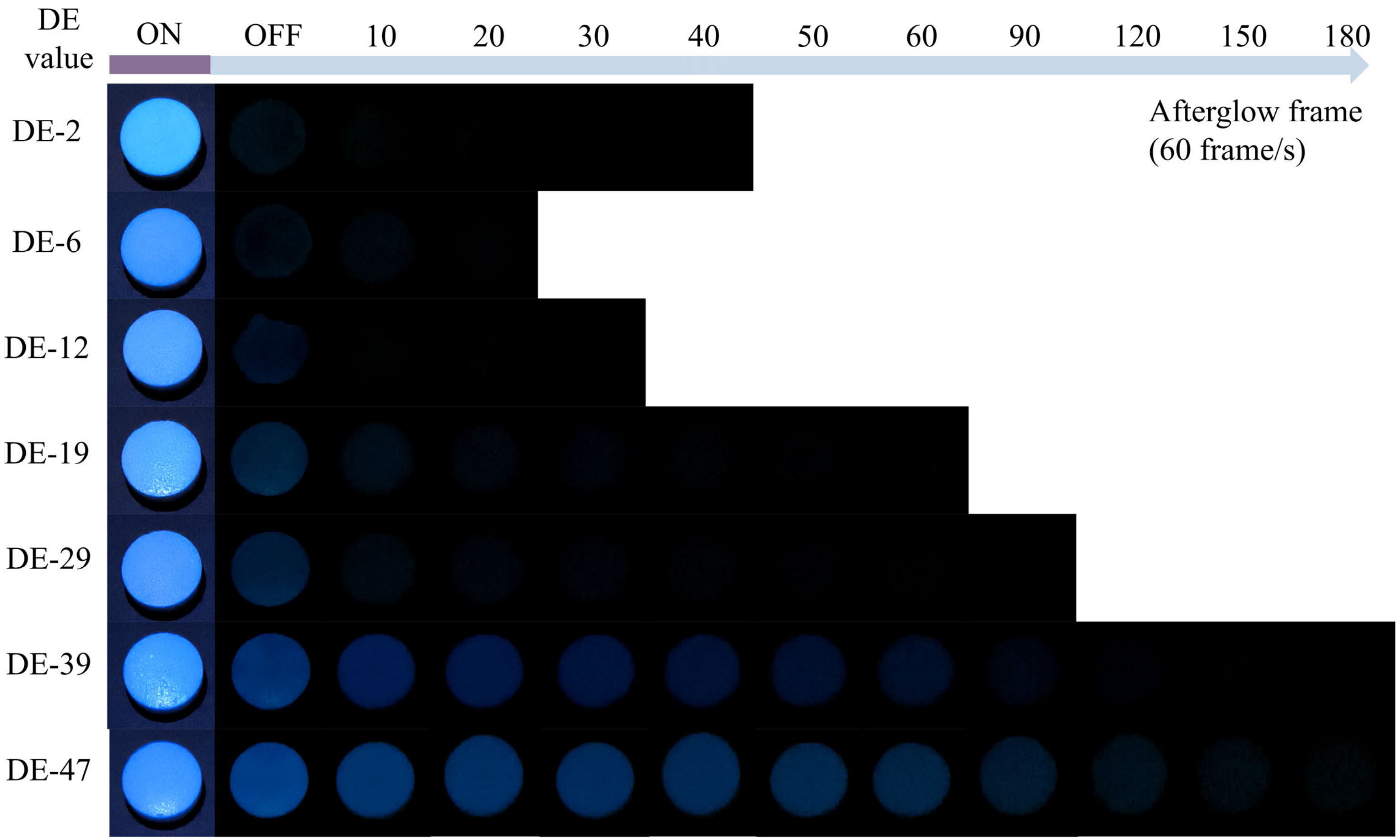

Figure S3. Afterglow photographs of MD tablet with different DE value stored in UV irradiation environment for 3 h.
